# Telomere-to-telomere genome assembly and 3D chromatin architecture of *Centella asiatica* insight into evolution and genetic basis of triterpenoid saponin biosynthesis

**DOI:** 10.1093/hr/uhaf037

**Published:** 2025-02-07

**Authors:** Wan-ling Song, Bao-zheng Chen, Lei Feng, Geng Chen, Si-mei He, Bing Hao, Guang-hui Zhang, Yang Dong, Sheng-chao Yang

**Affiliations:** State Key Laboratory of Conservation and Utilization of Bio-resources in Yunnan, The Key Laboratory of Medicinal Plant Biology of Yunnan Province, National-Local Joint Engineering Research Center on Germplasms Innovation & Utilization of Chinese Medicinal Materials in Southwest China, Yunnan Agricultural University, Kunming 650201, China; Yunnan Characteristic Plant Extraction Laboratory, Kunming, Yunnan 650106, China; College of Food Science and Technology, Yunnan Agricultural University, Kunming 650201, Yunnan, China; State Key Laboratory of Phytochemistry and Natural Medicines, Kunming Institute of Botany, Chinese Academy of Sciences, Kumming 650201, China; State Key Laboratory of Conservation and Utilization of Bio-resources in Yunnan, The Key Laboratory of Medicinal Plant Biology of Yunnan Province, National-Local Joint Engineering Research Center on Germplasms Innovation & Utilization of Chinese Medicinal Materials in Southwest China, Yunnan Agricultural University, Kunming 650201, China; Yunnan Characteristic Plant Extraction Laboratory, Kunming, Yunnan 650106, China; State Key Laboratory of Conservation and Utilization of Bio-resources in Yunnan, The Key Laboratory of Medicinal Plant Biology of Yunnan Province, National-Local Joint Engineering Research Center on Germplasms Innovation & Utilization of Chinese Medicinal Materials in Southwest China, Yunnan Agricultural University, Kunming 650201, China; Yunnan Characteristic Plant Extraction Laboratory, Kunming, Yunnan 650106, China; State Key Laboratory of Conservation and Utilization of Bio-resources in Yunnan, The Key Laboratory of Medicinal Plant Biology of Yunnan Province, National-Local Joint Engineering Research Center on Germplasms Innovation & Utilization of Chinese Medicinal Materials in Southwest China, Yunnan Agricultural University, Kunming 650201, China; Yunnan Characteristic Plant Extraction Laboratory, Kunming, Yunnan 650106, China; State Key Laboratory of Conservation and Utilization of Bio-resources in Yunnan, The Key Laboratory of Medicinal Plant Biology of Yunnan Province, National-Local Joint Engineering Research Center on Germplasms Innovation & Utilization of Chinese Medicinal Materials in Southwest China, Yunnan Agricultural University, Kunming 650201, China; Yunnan Characteristic Plant Extraction Laboratory, Kunming, Yunnan 650106, China; State Key Laboratory of Conservation and Utilization of Bio-resources in Yunnan, The Key Laboratory of Medicinal Plant Biology of Yunnan Province, National-Local Joint Engineering Research Center on Germplasms Innovation & Utilization of Chinese Medicinal Materials in Southwest China, Yunnan Agricultural University, Kunming 650201, China; Yunnan Characteristic Plant Extraction Laboratory, Kunming, Yunnan 650106, China; State Key Laboratory of Conservation and Utilization of Bio-resources in Yunnan, The Key Laboratory of Medicinal Plant Biology of Yunnan Province, National-Local Joint Engineering Research Center on Germplasms Innovation & Utilization of Chinese Medicinal Materials in Southwest China, Yunnan Agricultural University, Kunming 650201, China; Province Key Laboratory, Biological Big Data College, Yunnan Agricultural University, Kunming 650201, Yunnan, China; State Key Laboratory of Conservation and Utilization of Bio-resources in Yunnan, The Key Laboratory of Medicinal Plant Biology of Yunnan Province, National-Local Joint Engineering Research Center on Germplasms Innovation & Utilization of Chinese Medicinal Materials in Southwest China, Yunnan Agricultural University, Kunming 650201, China; Yunnan Characteristic Plant Extraction Laboratory, Kunming, Yunnan 650106, China; Honghe University, Mengzi, Yunnan 661199, China

## Abstract

*Centella asiatica* is renowned for its medicinal properties, particularly due to its triterpenoid saponins, such as asiaticoside and madecassoside, which are in excess demand for the cosmetic industry. However, comprehensive genomic resources for this species are lacking, which impedes the understanding of its biosynthetic pathways. Here, we report a telomere-to-telomere (T2T) *C. asiatica* genome. The genome size is 438.12 Mb with a contig N50 length of 54.12 Mb. The genome comprises 258.87 Mb of repetitive sequences and 25 200 protein-coding genes. Comparative genomic analyses revealed *C. asiatica* as an early-diverging genus within the Apiaceae family with a single whole-genome duplication (WGD, Apiaceae-ω) event following the ancient γ-triplication, contrasting with Apiaceae species that exhibit two WGD events (Apiaceae-α and Apiaceae-ω). We further constructed 3D chromatin structures, A/B compartments, and topologically associated domains (TADs) in *C. asiatica* leaves, elucidating the influence of chromatin organization on expression WGD-derived genes. Additionally, gene family and functional characterization analysis highlight the key role of *CasiOSC03* in α-amyrin production while also revealing significant expansion and high expression of CYP716, CYP714, and UGT73 families involved in asiaticoside biosynthesis compared to other Apiaceae species. Notably, a unique and large *UGT73* gene cluster, located within the same TAD, is potentially pivotal for enhancing triterpenoid saponin. Weighted gene coexpression network analysis (WGCNA) further highlighted the pathways modulated in response to methyl jasmonate (MeJA), offering insights into the regulatory networks governing saponin biosynthesis. This work not only provides a valuable genomic resource for *C. asiatica* but also sheds light on the molecular mechanisms driving the biosynthesis of pharmacologically important metabolites.

## Introduction

*Centella asiatica* (L.) Urban (2n = 2x = 18), commonly known as gotu kola, is a perennial herbaceous plant of the Apiaceae family, widely distributed in tropical and subtropical regions [1–3]. This species is renowned for its richness in triterpenoid saponins, which have been extensively studied for their therapeutic properties. In traditional medicine, particularly Traditional Chinese Medicine, *C. asiatica* has been utilized for centuries to treat a variety of ailments, including skin disorders, neurodegenerative diseases, and gastrointestinal conditions, etc. [4–8].

Among its bioactive constituents, pentacyclic triterpenoids and their saponins are particularly significant. Compounds such as asiaticoside, madecassoside, and their aglycones have garnered considerable attention due to their potential applications in medicine and cosmetics [9]. The biosynthetic pathway of these triterpenoids involves key processes such as carbon skeleton formation, hydroxylation for structural modifications, and glycosylation, among others. Studies have shown that the CYP716 family plays a crucial role in triterpene metabolism across various eudicot plants, particularly in the hydroxylation of α-amyrin at the C-28 position, a key step in the formation of the ursane-type triterpene saponin backbone [10–14]. Moreover, *C. asiatica-*specific members of the *CYP716* and *CYP714* gene families have been identified as critical for the hydroxylation process, underscoring their importance in saponin biosynthesis [15, 16]. Additionally, the UGT73 glycosyltransferase family is essential for the glycosylation of the C-28 position, further contributing to the complexity and specificity of triterpenoid saponin biosynthesis in *C. asiatica* [17–22]. Though the biosynthetic pathways of triterpenoid saponins are well characterized in some aspects, understanding of these pathways still remains incomplete, particularly regarding the characterization and regulatory mechanisms involving triterpenoid saponin-related genes that are unique to *C. asiatica* compared to other species within the Apiaceae family.

**Figure 1:**
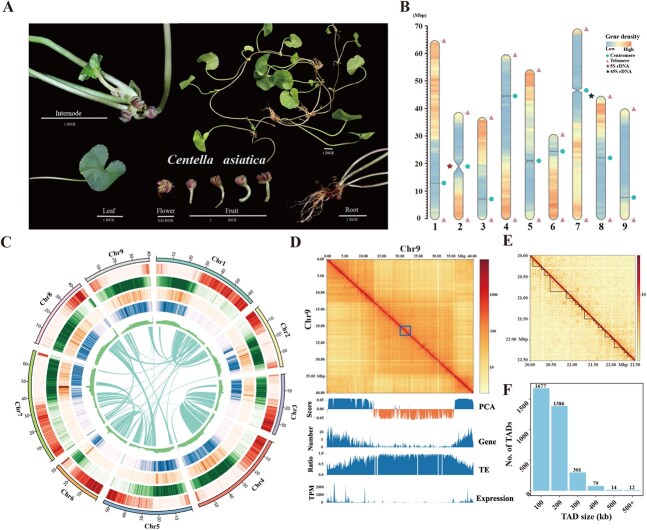
T2T wild *C. asiatica* genome. **A**. Phenotypic characteristics of different tissues of wild *C. asiatica.* All genomic data in our study were derived from leaves, while transcriptomic data were obtained from roots, stems, and leaves. **B**. Gene density and the distribution of telomeres and centromeres of *C. asiatica* genome. **C**. Genome characteristics of *C. asiatica*. The quantitative tracks are aggregated in 500-kb bins and the tracks from outside to inside are shown as follows: pseudochromosomes (Chr1 to Chr9), gene density, TE density, Gypsy LTR density, Copia LTR density, GC content, and chromosomal collinearity. **D**. Hi-C interaction matrix (100-kb resolution), A/B compartments (PCA score), gene density, TE density, and gene expression throughout chromosome 9. The Hi-C and RNA-seq data were derived from leaf tissues. **E**. Representative TAD structures in chromosome 9. . The 5-kb resolution was used for drawing Hi-C contact heatmap. **F**. Size distributions of topologically associated domains in the whole genome.

Recent advances in telomere-to-telomere (T2T) genomics have provided unprecedented insights into the genetics and evolution of new genes for specialized metabolism in plants [23–25]. However, the absence of a high-quality, T2T genome assembly for *C. asiatica* has limited our ability to fully elucidate the biosynthetic pathways and regulatory networks governing triterpenoid biosynthesis. The only available genome assembly for this species, derived from the cultivar ‘BB-174’, is fragmented and contains numerous gaps [26], hindering comprehensive genomic analysis. Meanwhile, the exploration of chromatin architecture provides a deeper understanding of gene regulation in plants. The 3D organization of the genome, including the formation of A/B compartments and topologically associated domains (TADs), plays a crucial role in regulating gene expression by influencing the spatial arrangement of chromatin and the accessibility of transcriptional machinery [27, 28]. In *C. asiatica*, understanding the 3D genome structure is particularly important for elucidating how chromatin organization affects the expression of duplicated genes and other key genes involved in specialized metabolism. Despite recent advances in Hi-C technology, which have allowed for the mapping of 3D genome structures in various plant species, the specific role of chromatin architecture in regulating gene expression in *C. asiatica* remains largely unexplored.

In light of these gaps, we present the first complete T2T genome assembly of *C. asiatica*, which was achieved through a combination of Illumina, PacBio HiFi, and Hi-C technologies. Based on the assembled T2T genome, we constructed the 3D chromatin structure and investigated how the expression levels of homologous gene pairs originated from whole-genome duplication (WGD) are influenced by this chromatin organization. Additionally, we characterized the evolutionary dynamics and functional characterization of key gene families involved in triterpenoid biosynthesis, including oxidosqualene cyclases (OSCs), cytochrome P450 (CYP450s), and UDP glycosyltransferases (UGTs). Following methyl jasmonate (MeJA) treatment, gene expression profiles and coexpression networks were constructed. Our integrative analysis, combining genome and transcriptome data, provides new insights into the regulation of triterpenoid biosynthesis and offers a valuable genomic resource for future research on the evolutionary dynamics and genetic breeding of *C. asiatica*.

## Results

### The T2T gap-free genome assembly, quality evaluation, and annotation of *C. asiatica*

In this study, we assembled the genome of wild *C. asiatica* (Fig. 1A) via a combination of Next Generation Sequencing (NGS) reads (50.55 Gb ~114×), PacBio HiFi long reads (21.45 Gb ~ 48×), and Hi-C data (88.22 Gb ~198×) (Table S1). K-mer analysis (k = 17) indicated that the genome is diploid with an estimated size of 440.69 Mb and a heterozygosity of 0.038% (Fig. S1A and B). The initial assembly using HiFi reads produced a 444.25Mb genome (Fig. S1C), which was further anchored into nine pseudochromosomes via Hi-C data. Among these, four pseudochromosomes are gap-free, while three others have minimal gaps (Table S2). Unanchored contigs were identified as redundant sequences and chloroplast or mitochondrial DNA. Gaps within pseudochromosomes were closed with the GapFiller module, resulting in a final genome size of 438.12 Mb with a scaffold N50 length of 54.12 Mb and a GC content of 34.21%. With the repeated sequence of ‘AAACCCT’ as the basis for telomere detection, telomeres were identified at the ends of all pseudochromosomes, with lengths ranging from 1353 to 6521 bp (Fig. 1B; Table S3).

Additionally, the centromeres were located via the CentroMiner module and ranged in length from 8 to 4820 kb (Fig. 1B; Table S4). The large centromeres, >1 Mb in length, were found on chromosomes 2 and 7, and were validated by Hi-C interaction signals (Fig. S2A and B) and pairwise sequence identity plots (Fig. S2C and D). Further analysis of transposable elements in the centromeric regions revealed that 56.59% of the centromeric regions are occupied by long terminal repeat sequences (LTRs), with Gypsy being the predominant LTR type, accounting for 77%, suggesting that Gypsy-type LTRs have invaded the centromeric regions and may play a key role in shaping the structure of the centromeres in *C. asiatica*. By comparing LTR insertion times in centromeric regions with those in noncentromeric regions, we observed that LTR sequences in centromeric regions are relatively young (Fig. S2E), which may drive the evolution of centromeres in *C. asiatica*. Additionally, a substantial 5S rDNA array located in centromeric regions was identified on chromosome 2 (16.79–21.32 Mb) (Fig. S1D). Meanwhile, a 45S rDNA array spanning ~461.36 kb was annotated at the terminus of chromosome 8 (43 830 112–44 291 472 bp), which is in close proximity to the telomere (Fig. S1D). This assembly represents a highly complete, accurate, and continuous T2T genome of wild *C. asiatica* (Fig. 1C).

The completeness and accuracy of the genome were further confirmed by mapping NGS reads, resulting in a mapping rate exceeding 99% with even distribution across the genome (Fig. S3; Table S5). The genome achieved a QV value of 44.45, with k-mer completeness of 99.21% and base accuracy of 99.99996%. Compared with the previously reported cultivated *C. asiatica* BB-174 genome, our assembly shows substantial improvements. Specifically, our wild genome’s contig N50 is 54.12 Mb, which is 832.6 times larger than the 0.065 Mb contig N50 of BB-174 (Table 1). The LTR Assembly Index (LAI) value also increased to 16.36, where it was 6.70 for BB-174. The assembly’s Benchmarking Universal Single-Copy Orthologs (BUSCO) completeness reached 99%, which was significantly higher than that of BB-174 (Fig. S4). Hi-C interaction heatmaps confirmed the accuracy of the pseudochromosome assembly (Fig. S5). Moreover, large-inversion structural variations were identified on chromosomes 1, 4, 5, 6, 7, and 8 of wild *C. asiatica*, which were further validated by Hi-C data (Fig. S6). These results underscore the superior completeness and continuity of our T2T genome compared with BB-174.

**Table 1:** Statistics for assembly and annotation of the *C. asiatica* genomes.

*C. asiatica* genome	Wild	BB-174 [26]
Genome size/Mb	438.12	430.217
GC level	34.21%	34.17%
No. of contigs	13	8739
Contig N50/Mb	54.12	0.065
No. of gaps	0	12 777
Telomere	18	\
Centromeres	9	\
LAI	16.36	6.70
No. of genes	25 200	25 226
Repeat Proportion	59.09%	56.38%

Based on the assembled genome, gene annotation was conducted by combining evidence from mRNA sequencing data generated from three tissues (leaf, stem, and root), protein sequences, and *ab initio* prediction. By repetitive sequence analysis, the *C. asiatica* genome was shown to contain 258.87 Mb of repetitive elements, covering 59.09% of the genome. Among them, Copia and Gypsy LTR retrotransposons were the most predominant repeat types, accounting for 26.04% and 16.50%, respectively, of the genome. This was followed by DNA transposons at 8.11% (Table S6). Additionally, 1641 Copia and 1047 Gypsy complete LTR retrotransposons were identified, which are 4.1 and 6.6 times greater than those found in the BB-174 genome, respectively (Fig. S4). The greater number of complete LTRs further demonstrates the quality of our assembly.

A total of 25 200 protein-coding genes were annotated via *ab initio*, homology-based, and transcript-based approaches. Gene function annotation of *C. asiatica* was subsequently performed, and an overall total of 23 711 functional genes were annotated, accounting for 94.09% of the total number of genes (Table S7). The BUSCO assessment of the annotated proteins suggested that ~97.80% of the BUSCOs were complete. Noncoding RNA analysis revealed the presence of 88 microRNAs, 9976 rRNAs, 367 tRNAs, 294 snRNAs, and 14 snoRNAs in the genome (Table S8).

### Orthologous clustering, phylogenetic analysis, and WGD assessment of *C. asiatica*

To elucidate the evolutionary trajectory of *C. asiatica*, a comparative genomics analysis was conducted with 16 representative plant species. Among these, nine species are from the Apiaceae family, encompassing *Daucus carota*, *Oenanthe javanica*, *Angelica sinensis*, *Apium graveolens*, *Coriandrum sativum*, *Oenanthe sinensis*, *Heracleum sosnowskyi*, *Notopterygium incisum*, and *Ligusticum chuanxiong*. Additionally, four species from the Araliaceae family were included, namely *Panax notoginseng*, *Eleutherococcus senticosus*, *Aralia elata*, and *Panax vietnamensis.* A gene family clustering analysis across all species revealed a total of 51 212 orthogroups, of which 3284 were prevalent in 17 species. The *C. asiatica* genome included 16 906 orthogroups and 22 612 genes (Table S9), accounting for 89.7% of the total number of genes. In comparison, an average of 88.4% of the genes from the other 16 plants were located in the orthologous cluster. Compared with the other 16 plants, *C. asiatica* presented a greater proportion of genes in orthologous clusters and a greater number of species-specific genes, although the total number of genes was lower. This may be related to the evolutionary history or adaptive strategies of the species. A comparison of Apiaceae species revealed that *C. asiatica* had the greatest number of single-copy genes (49.9%) (Fig. S7A), which means that *C. asiatica* has a relatively lower abundance of multicopy genes. The 188 gene families specific to *C. asiatica* contained a total of 502 genes (Fig. S7B and Table S10). Interestingly, two unique *UGT73* genes—*UGT73AD1* (*Cas03G001714*) and *UGT1* (*Cas03G001713*)—belong to those specific gene families associated with the biosynthesis of asiaticoside and madecassoside, key compounds in *C. asiatica* [21, 22]. While collinearity analysis revealed that 41.48% (in *N. incisum*) to 62.05% (in *O. sinensis*) of *C. asiatica* genes are syntenic with those of other Apiaceae species (Table S11), the chromosomal rearrangements were observed among these species (Fig. S8).

**Figure 2:**
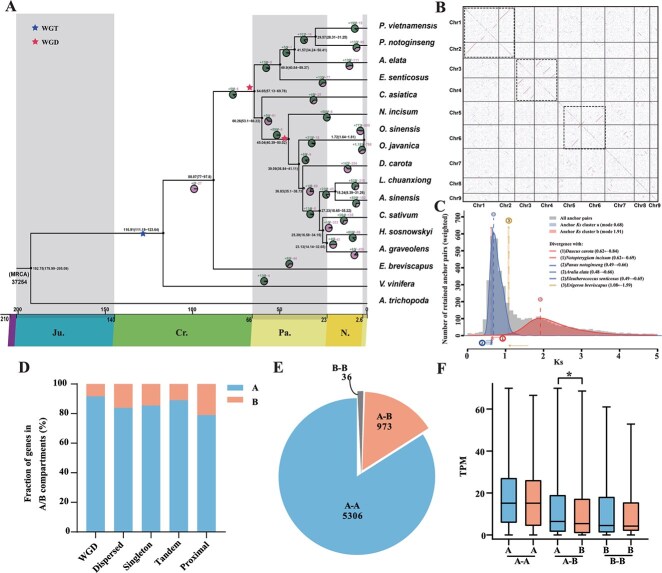
Phylogenetic and WGD analysis of *C. asiatica*. **A**. The ML phylogenetic tree for *C. asiatica* and 16 other species. The expansion and contraction of gene families are indicated with plus and minus signs, respectively. The time on the tree node represents the divergence time supported by 95% of the highest posterior density. The coordinates on the bottom are geological time (below) and absolute age (upper), with millions of years. MRCA, the most recent common ancestor. **B**. Self-syntenic dot plots within *C. asiatica* genome. Regions marked by dotted box are duplicated areas. **C**. Rate-adjusted mode estimates of one-to-one ortholog Ks distributions with *C. asiatica* as the focal species. The background represents the whole-paranome Ks distribution and the anchor-pair Ks distribution for *C. asiatica*. The vertical dashed lines labeled 'a' and 'b' denote the modes of these components, which serve as the basis for WGD age estimates. The rate-adjusted mode estimates of ortholog distributions between *C. asiatica* and other species, symbolizing speciation events, are depicted as numbered vertical long dashed lines. The horizontal arrows at the bottom of the plot illustrate the shifts in the speciation lines due to ksrates' substitution rate adjustments, with the corresponding value shifts detailed in the panel legend. **D**. Percentages of different duplicate types in A/B compartments. **E**. Count of WGD gene pairs located in the same and switch compartments. A-A' or 'B-B′ denotes that both gene pairs of the WGD genes are located within the same A or B compartment, respectively. Conversely, 'A-B′ signifies that the WGD genes are positioned in distinct compartment types. **F**. Comparison of the expression levels of WGD genes located in the same or switched A/B compartment. * indicates *P* < .05 (Wilcoxon rank-sum test).

Phylogenetic analysis of 1014 low-copy genes (76.5% of species having a single copy in this study) identified by OrthoFinder [29] from the 17 species revealed that *C. asiatica* is a genus within the Apiaceae family, diverging from other Apiaceae species ~53.1–66.22 million years ago (Fig. 2A). Gene family expansion and contraction analyses indicated that 46 gene families in *C. asiatica* underwent significant expansion, whereas only 25 gene families experienced contraction (Table S12). These expanded gene families were significantly enriched in Kyoto Encyclopedia of Genes and Genomes (KEGG) pathways such as stilbenoid, diarylheptanoid, gingerol biosynthesis, glycolysis/gluconeogenesis, and phenylpropanoid biosynthesis (Table S13). Additionally, a recent burst of Long terminal repeat-retrotransposons (LTR-RTs) was detected in *C. asiatica* ~57 000–86 000 years ago, similar to other Apiaceae species except *A. sinensis* (Fig. S9A). The amplified LTR retrotransposons were predominantly from the Copia and Gypsy families, which may have contributed to the genomic changes observed in *C. asiatica* (Fig. S9B).

WGD events have been pivotal in the genomic evolution of *C. asiatica*. Among the 25 200 genes in *C. asiatica*, 6315 duplicated gene pairs, comprising 9364 genes (37.16% of all genes), are distributed across 334 collinear blocks. Genomic dot plot analysis revealed a 1:2 syntenic relationship within *C. asiatica*, with significant collinearity observed, particularly between Chr1 and 2, Chr3 and 4, and Chr5 and 6 (Fig. 2B). These findings suggest that *C. asiatica* may have undergone a WGD event. A rate-adjusted synonymous substitution rate (Ks) analysis further corroborated this finding, revealing a distinct peak (Ks = 0.68) indicative of a WGD event occurring after the ancient γ-triplication event (Fig. 2C). To determine whether this WGD event was shared by the Apiaceae family or the Apiales order, we used the rate-adjusted Ks distribution, revealing that the WGD event in *C. asiatica* was shared by Apiales, rather than only in Apiaceae (Fig. 2C). Furthermore, we have provided further evidence of collinearity. By selecting *C. asiatica* (from the Apiaceae family) and *A. elata* (from the Araliaceae family) as representatives, we observed a clear 2:1 ratio of collinear relationships with *Vitis vinifera* (from the Vitaceae family), suggesting that both species underwent a tetraploidization event following their divergence from Vitaceae (Fig. S10). The collinearity relationship between *C. asiatica* and *A. elata* is 2:2, further supporting the presence of similar WGD events between these two species (Fig. S10). When comparing species from the Apioideae subfamily with *C. asiatica*, we found a 2:4 ratio of collinear relationships (Fig. S8), indicating that other species within the Apiaceae family shared a novel WGD (Apiaceae-α) relative to *C. asiatica*. The Ks peak plots for other species within the Apiaceae family also support this finding (Fig. S11). In addition, other species from Apiaceae family, such as *N. incisum* compared with the *V. vinifera* genome, presented a 4:1 ratio of collinear relationships (Fig. S10), which further confirms the aforementioned conclusions. These results collectively indicate that *C. asiatica* has undergone a shared WGD event (Apiaceae-ω) within the Apiales, but lacked the recent Apiaceae-α WGD event, which is consistent with conclusions drawn via the OI method [30]. The reduced gene redundancy highlights its potential as a model for studying specific gene functions and evolutionary dynamics within the Apiaceae family.

### Three-dimensional chromatin structure and its influence on expression of WGD-derived genes in *C. asiatica*

The spatial organization of the genome is essential for regulating gene expression. Using 311.83 million Hi-C read pairs (Table S14), we constructed a 3D chromatin map of the *C. asiatica* genome. Interaction Decay Exponent (IDE) analysis revealed a sharp decline in interaction signals as genomic distance increased, with an average exponent of −0.97 across all chromosomes. In line with previous studies on plant genomes, we identified A/B compartments and TADs in *C. asiatica*. The A compartment encompasses 234.6 Mb of genomic sequences (53.55% of the genome), characterized by higher gene density and transcriptional activity, while the B compartment, covering 175.93 Mb (40.165%), is enriched with transposable elements (TEs) (Table S15, Fig. S12, and Fig. S13). From the 5-kb Hi-C interaction matrices, we identified 3469 TADs (Fig. 1E) with a median length of 105 kb (Table S16, Fig. S14), which were utilized for gene cluster analysis.

To elucidate the role of chromatin architecture in the expression divergence of genes during diploidization, we classified genes into five categories: WGD genes (37.16%), dispersed duplication (DSD) genes (34.86%), tandem duplication (TD) genes (6.20%), proximal duplication (PD) genes (2.54%), and single-copy (SC) genes (19.24%) (Table S17). WGD genes were predominantly located in the A compartments, with a proportion of 91.61% (Fig. 2D). Among the 6315 gene pairs from WGD, 2285 pairs were differentially expressed genes (DEGs) in leaf tissues (Fig. S15), suggesting expression divergence among WGD genes. Notably, 15.41% (973 out of 6315) of the gene pairs were located in different types of A/B compartments (Fig. 2E). Genes in the A compartments exhibited significantly higher expression levels than those in the B compartments, while no significant expression bias was observed for genes within the same A/B compartment type (Fig. 2F). Additionally, 186 DEGs, potentially regulated by chromatin status, were identified in compartment switch regions (Table S18). Among these genes, four genes are potentially associated with the biosynthesis of triterpenoid saponins. Two of these genes are part of the CYP family: the highly expressed *Cas05G001988* located in compartment A and *Cas03G000178* in compartment B. The remaining two genes, *Cas07G002524* and *Cas03G000219*, belong to the *UGT73* gene family, which is crucial for asiaticoside saponin biosynthesis in *C. asiatica*. Specifically, *Cas07G002524* is highly expressed in compartment A, while *Cas03G000219* exhibits is expressed at lower levels in compartment B. KEGG analysis of these 186 DEGs revealed that these DEGs are enriched in several pathways, such as carbon fixation in photosynthetic organisms and diterpenoid biosynthesis (Fig. S16).

**Figure 3:**
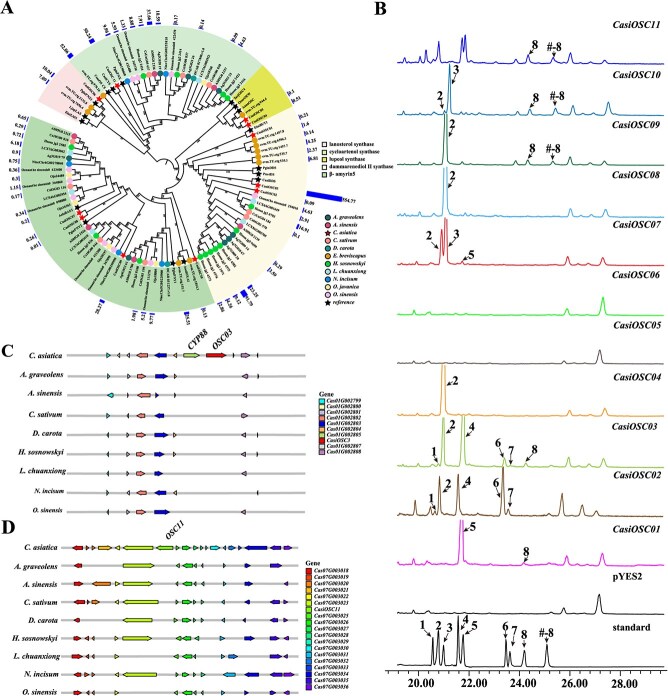
Evolution of *OSC* gene families of *C. asiatica*. **A.** ML phylogenetic tree of the *OSC* gene family in 11 species. The number on the branch represents the bootstrap value, the same graph is used at each terminal branch to represent the same species, and the outermost bar indicates the expression amount of each gene in the leaves. **B**. Functional characterization of *CasiOSCs* via heterologous expression. Compounds 1–8 were identified as δ-amyrin, β-amyrin, Germanicol, α-amyrin, lanosterol, ψ-taraxasterol, taraxasterol, and dammarendiol-II. The hash (#) in the total ion chromatograms (TICs) represents dammarenediol-II mono-trimethylsilyl. **C.** Schematic organization of the *OSC03* gene distribution surrounding *C. asiatica* in nine Apiaceae species. Another gene *CYP88* was also identified. The illustration only showed the homologs of genes within the collinear regions between *C. asiatica* and other Apiaceae species. The length of the arrow represents the length of the gene. **D**. Schematic organization of the *OSC11* gene distribution surrounding *C. asiatica* in nine Apiaceae species.

### Evolutionary dynamics and functional characterization of the *OSC* gene family in *C. asiatica*

Metabolomic profiling of *C. asiatica* leaves identified 99 terpenoid compounds, including diterpenoids (3), monoterpenoids (26), sesquiterpenoids (9), and triterpenoids (61). Among these ones, two tetracyclic triterpenoids (cycloartanol and isomangiferolic acid) and 45 pentacyclic triterpenoids were detected, along with 14 triterpenoid saponins predominantly of the ursane (α-) and oleanane (β-) types (Table S19). The OSCs play a crucial role in catalyzing the formation of diverse triterpene aglycone skeletons [31]. In *C. asiatica*, we identified 11 *OSC* genes (Table S20), with *CasiOSC03* (*Cas01G002806*), previously implicated as a key gene in the formation of saponin skeletons [10, 16, 31], exhibiting the highest expression levels in leaves (Fig. S17). Additionally, *CasiOSC11* (*Cas07G003024*) and *CasiOSC09* (*Cas07G002173*) were expressed in stems and leaves, while *CasiOSC06* (*Cas03G000009*) was predominantly expressed in roots. The remaining genes showed minimal or no expression across the tissues examined (Fig. S17). Gene cluster analysis using plantiSMASH identified two clusters involved in terpene biosynthesis (Table S21). Cluster 3, located at Chr1:48985198-49 020 888, included *CasiOSC03*, a CYP88 family gene (*Cas01G002805*), and a BAHD family gene (*Cas01G002804*). Cluster 22, located at Chr7:32238972-32 550 726, contains *CasiOSC11*, two dioxygenase genes (*Cas07G003031, Cas07G003033*), one COesterase gene (*Cas07G003028*), and one CYP704 gene (*Cas07G003020*). The gene organization in Cluster 22 is not conserved between *C. asiatica* and other Apiaceae species. These two terpene clusters suggest that *CasiOSC03* and *CasiOSC11* may be involved in terpene biosynthesis by gene clusters in *C. asiatica*.

Comparative analysis of *OSC* genes in other Apiaceae species revealed that *H. sosnowskyi* has the greatest number of OSC members (14), followed by *C. asiatica* (11), with *D. carota* having the fewest (2) (Table S20). WGD and tandem duplication events were identified as the primary mechanisms driving *OSC* gene expansion in Apiaceae (Fig. S18). Phylogenetic analysis grouped the *OSC* genes into five distinct clusters (Fig. 3A). *CasiOSC03, CasiOSC01*, and *CasiOSC02* clustered within the dammarenediol II synthase (DDS) branch, while *CasiOSC07, CasiOSC08, CasiOSC09,* and *CasiOSC10* were classified into the β-amyrin branch. *CasiOSC06* and *CasiOSC11* were categorized under the lanosterol synthase and cycloartenol synthase branches, respectively, while *CasiOSC04* and *CasiOSC05* were grouped with lupeol synthase (Fig. 3A).

We have amplified and constructed *pYES2-CasiOSC01* ~ *CasiOSC11* expression vectors and further fermented in the mutant yeast strain GIL77 for functional characterization. Five products were identified in *CasiOSC02* and *CasiOSC03* (Fig. 3B, Figs S19–S22)*.* However, the activity of *CasiOSC03* to catalyze the formation of α- amyrin **(4)** from 2,3-oxidosqualene was significantly higher than that of *CasiOSC02*, and trace amounts of dammarenediol II **(8)** were detected in strains *CasiOSC01, 03* and *09, 10, 11*, which demonstrated that these *OSC* genes catalyze the formation of dammarenediol II. The β-amyrin **(2)** was identified as the main product of *CasiOSC04*, *07, 08*, and *09.* Surprisingly, germanicol and lanosterol were identified in *CasiOSC07*, *CasiOSC10,* and *CasiOSC01*, respectively, while *CasiOSC11* showed the production of cycloartenol (Fig. S23). Unfortunately, no triterpenoid peaks were detected in *CasiOSC05* or *CasiOSC06.* Additionally, the functional characterization of *CasiOSCs* reveals that α- and β-amyrin are the predominant products, aligning with the observed accumulation pattern of pentacyclic triterpenoids in *C. asiatica*. Collinearity analysis revealed that the highly expressed genes *CasiOSC03* and *CasiOSC11* were lost or rearranged in other Apiaceae species (Fig. 3C and D), suggesting that the key *OSC* genes involved in specialized biosynthesis of asiaticoside saponins in *C. asiatica* are not conserved within the Apiaceae family.

### Expansion and evolutionary divergence of the *CYP450* gene family in *C. Asiatica*

CYP450 enzymes play a pivotal role in hydroxylation and other modification reactions involved in triterpenoid saponin biosynthesis, which directly impact the diversity and biological activity of these compounds in *C. asiatica* [16]. Given that nearly half of the enzymatic reactions in this pathway are mediated by CYP450s, a comprehensive understanding of these enzymes is essential for elucidating the biosynthesis of compounds in *C. asiatica*. Using the HMM model (PF00067), we identified 160 *CYP450* genes in *C. asiatica* (Table S22.). Phylogenetic analysis, based on sequence comparisons with homologous genes from the NCBI database and P450Rdb [32], classified these genes into 39 subfamilies, including CYP81, CYP82, CYP714, and CYP716, etc. (Fig. S24). Most subfamilies, with the exception of CYP82 and CYP81, contain <10 genes, and six subfamilies are represented by only a single gene (Fig. S24).

**Figure 4:**
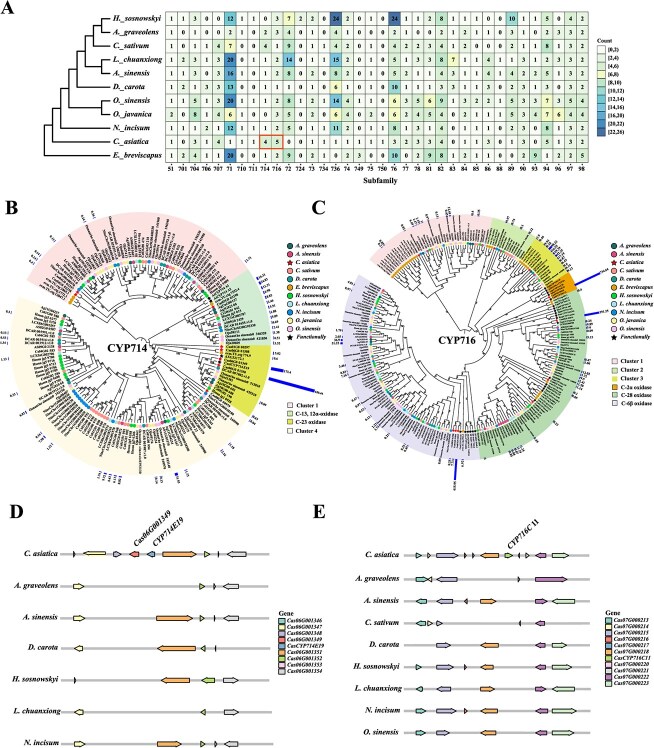
Evolution of *CYP450s* gene families of *C. asiatica*. **A**. Phylogenomic analysis of the CYP450s in the 11 plant species. The color of each block is based on the number of highly expressed genes (TPM > 5) in each subfamily. **B**. ML phylogenetic tree of the CYP714 subfamily in 11 species. **C**. ML phylogenetic tree of the CYP716 subfamily in 11 species. The number on the branch represents the bootstrap value, the same graph is used at each terminal branch to represent the same species, and the outermost bar indicates the expression level of each gene in the leaves. **D**. Schematic organization of the *CYP714E19* gene distribution surrounding *C. asiatica* in seven Apiaceae species. Another gene *Cas06G001349* from the UGT714 subfamily was also identified. The illustration only focused on the homologous genes within the collinear regions between *C. asiatica* and other Apiaceae species. The length of the arrow represents the length of the gene. **E**. Schematic organization of the *CYP716C11* gene distribution surrounding *C. asiatica* in nine Apiaceae species.

Comparative analysis with nine species from the Apiaceae family and *Erigeron breviscapus* from Asteraceae revealed that *C. asiatica* harbored the fewest number of *CYP450* genes among the species analyzed (Fig. S25), suggesting a contraction of this gene family during its evolutionary history, likely due to the loss of the Apiaceae-α WGD event. Further analysis of gene duplication types across these species showed that *C. asiatica* also had the fewest genes arranged in tandem (Fig. S26). Despite this, focusing on highly expressed genes (TPM > 5) in leaves, we observed an expansion of the CYP714 and CYP716 families in *C. asiatica* (Fig. 4A). Notably, WGD is the predominant duplication type, with nearly all duplicated genes arising from WGD, except for a single tandem gene in the CYP714 family (Fig. S27). Known genes such as *CYP716A83* (*Cas04G002074*), *CYP716C11* (*Cas07G000219*), *CYP716E41* (*Cas07G002218*), and *CYP714E19* (*Cas06G001350*) play critical roles in the asiaticoside biosynthetic pathway, all belonging to the CYP714 and CYP716 subfamilies [11, 16]. This expansion likely contributes to the biosynthesis of asiaticoside.

Phylogenetic analysis of the CYP714 and CYP716 subfamilies across 11 species revealed that the genes clustered into six and four groups, respectively, each associated with distinct catalytic functions (Fig. 4B and C). Notably, the *CYP714* genes involved in asiaticoside biosynthesis were clustered into the second and third groups. Focusing on a tandem gene cluster that includes the known gene *CYP714E19* and its tandem repeat *Cas06G001349* (Fig. 4D), both genes exhibited high expression levels in leaf tissues (Fig. 4B). Comparative genomic analysis of the region surrounding *CYP714E19* in Apiaceae species indicated the absence of homologous *CYP* genes in other species (Fig. 4D). Similarly, the most highly expressed genes in the CYP716 subfamily, including *CYP716E41* and *CYP716C11*, also lacked homologous sequences in the collinear regions of other Apiaceae species (Fig. 4E). These results suggest that the key *CYP* genes associated with the specialized biosynthesis of asiaticoside in *C. asiatica* are not evolutionarily conserved within the Apiaceae family.

### Expansion and distribution of the *UGT73* gene cluster in asiaticoside saponin biosynthesis of *C. asiatica*

UGT glycosyltransferases are essential for the biosynthesis of C-28 glycosylated triterpenoid saponins in *C. asiatica*. A total of 117 *UGT* genes were identified in the *C. asiatica* genome and categorized into known subfamilies, including UGT73, UGT71, UGT94, UGT91, UGT85, and UGT74 (Fig. S28 and Table S23). The primary duplication types for *UGT* genes in *C. asiatica* are tandem repeats and WGD, with tandem duplications accounting for 34.19%, which is notably higher than those in other species (10.53%–27.13%) (Fig. S29). In comparative analysis with other Apiaceae species, no expanded subfamily was observed in *C. asiatica* (Fig. S30).

**Figure 5:**
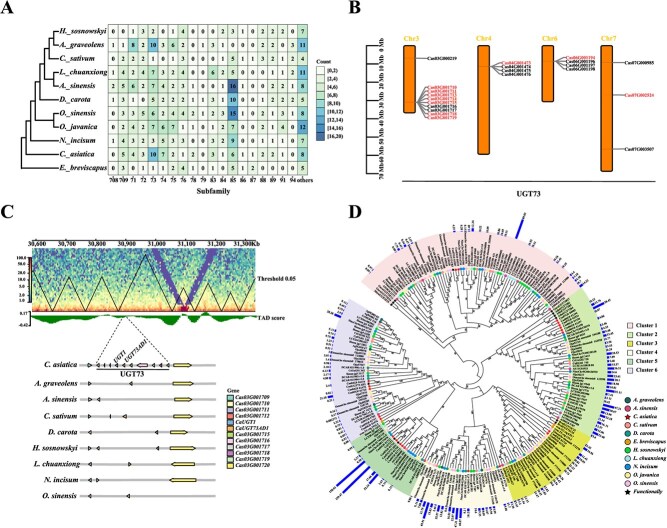
Evolution and genomic organization of *UGT73* gene families of *C. asiatica*. **A**. Phylogenomic analysis of the UGTs in the 11 plant species. The color of each block is based on the number of highly expressed genes (TPM > 5) in each subfamily. **B**. Map of the location distribution of UGT73 subfamily genes on the chromosomes of *C. asiatica*. **C**. *UGT73* gene cluster present in *C. asiatica*. The *UGT73* gene cluster of *C. asiatica* and its nearby 3D genomic chromatin maps are shown. A schematic organization of these gene distributions in seven species was drawn and the known genes that participate in the biosynthesis of asiaticoside were labeled. The arrow length and direction represent the relative length and direction of the gene. **D**. ML phylogenetic tree of the UGT73 subfamily in 11 species. The number on the branch represents the bootstrap value, the same graph is used at each terminal branch represent the same species, and the outermost bar indicates the expression level of each gene in the leaves.

Focusing on highly expressed *UGT* genes (TPM > 5) in leaves, we observed an expansion of the UGT73 subfamily in *C. asiatica* (Fig. 5A). Within this subfamily, tandem repeats are predominant, accounting for 66.67% of the genes, in contrast to the WGD duplication observed in other species (Fig. S31). Chromosomal mapping showed a pronounced uneven distribution of *UGT73* genes, with a significant presence of tandem repeats on chromosomes 3, 4, and 6 (Fig. 5B). Notably, 10 *UGT73* genes are located on chromosome 3, where nine genes form a prominent tandem repeat cluster. Seven of these genes exhibited high expression levels (Fig. 5B). All the *UGT73* genes within this tandem cluster are located in the same TAD (TAD1044, Chr3: 30875000-31 055 000) as determined by 3D genomic analysis (Fig. 5C and Table S11). This spatial organization suggests coordinated regulatory functions among these *UGT73* genes. Collinearity analysis conducted across nine Apiaceae species has unveiled that other species typically harbor either one or two copies of the *UGT73* genes (Fig. 5C). Phylogenetic analysis classified *UGT73* genes into six distinct groups, each with specific biocatalytic functions (Fig. 5D). Notably, previously reported genes associated with asiaticoside biosynthesis, including *UGT73AH1* (*Cas04G001474*), *UGT73AD1* (*Cas03G001714*), and *UGT1* (*Cas03G001713*), are clustered in Groups 5 and 6 [17, 18], while the remaining genes *Cas03G001710*, *Cas03G001711*, *Cas03G001715*, *Cas03G001717*, *Cas04G001475*, and *Cas04G001476* are distributed across Groups 5 and 6. Among them, the genes *Cas03G001710*, *Cas03G001711*, *Cas03G001715*, and *Cas03G001717*, which belong to the tandem repeat cluster on Chr3, are highly expressed. These findings suggest that the presence of *UGT73* gene cluster and high transcriptional activity may contribute significantly to the enhancement of triterpenoid saponin biosynthesis in *C. asiatica*.

### Gene expression profiling and coexpression network analysis following MeJA treatment in *C. asiatica*

**Figure 6:**
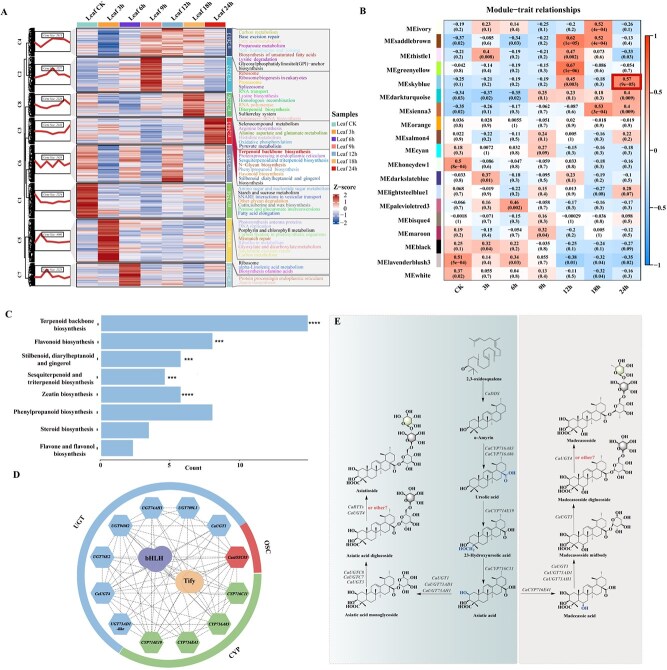
Characterization of genes associated with asiaticoside and madecassoside biosynthetic pathways and their expression patterns in response to MeJA. **A**. Gene clusters C1–C8 identified through Mfuzz analysis of leaf transcriptomes at time points 0,3, 6, 9, 12, 18, and 24 h under MeJA treatment. The enriched KEGG pathways for each cluster were annotated based on a significance level of *P* < .05. **B**. WGCNA analysis of the transcriptome data at six time points after MeJA treatment. **C**. KEGG enrichment of genes in the ‘skyblue’ module. **D**. A coexpression network connecting possibly OSCs, CYPs, and UGTs in asiaticoside and madecassoside biosynthesis with TFs in the ‘skyblue’ module. The nodes represent structural genes involved in asiaticoside and madecassoside biosynthesis and TFs and the lines indicate interactions between the two genes. **E**. Biosynthetic pathways and structural genes of asiaticoside and madecassoside.

Following MeJA treatment, leaf and stem tissues were collected at 0, 3, 6, 9, 12, 18, and 24 h for transcriptome sequencing. The genes expressed in these tissues were clustered into eight groups via K-means clustering (C1–C8) (Fig. 6A). To elucidate the biological functions of these clusters, KEGG enrichment analysis was performed, revealing significant enrichment of the Terpenoid Backbone Biosynthesis pathway in Cluster C3 from leaves, which also showed a consistent upregulation at 24 h post-treatment (Fig. 6A). These findings suggested that these genes related to triterpenoid biosynthesis were particularly activated at 24 h under MeJA treatment. In contrast, no significant enrichment in triterpene biosynthesis pathways was observed in the stem tissue (Fig. S32). To further understand the relationship between metabolite accumulation and gene expression in *C. asiatica*, we performed Weighted Gene Co-expression Network Analysis (WGCNA) and identified 19 coexpression modules (Fig. S33). A heatmap of gene expression across treatment times highlighted a significant positive correlation for 109 genes within the ‘skyblue’ module at 24 h (Fig. 6B). This module was notably enriched in pathways related to terpenoid backbone biosynthesis, flavonoid biosynthesis, and both sesquiterpenoid and triterpenoid biosynthesis (Fig. 6C), which further supports that the pivotal time for actively initiating triterpenoid biosynthesis occurs at 24 h under MeJA treatment.

Further analysis of the ‘skyblue’ module revealed 14 genes associated with triterpenoid biosynthesis (Table S24), including one *OSC* gene (*CasiOSC03*), two known *CYP714* genes (*CYP714E19*, *CYP716E41*), two known *CYP716* genes (*CYP716A83*, *CYP716C11*), two newly identified *CYP94* genes (*CYP94C11*5, *CYP94C1*), and seven *UGT* genes, comprising two known genes (*UGT1* and *CaUGT4*) and five new genes (*UGT709L1, UGT74AH1, UGT94M2, UGT76E2*, and *UGT73_AD1*-like). Additionally, five classes of transcription factors (TFs)—bHLH, MYB, MYB-related, NAC, and TIFY—were identified in this module (Table S25.). Correlation analysis of these genes indicated interactions between these TFs and the 14 key genes, suggesting their role as activators in the regulation of asiaticoside and madecassoside biosynthesis (Fig. 6D).

Building on previous studies and the results presented above [16, 20, 33, 34], the biosynthetic pathways for asiaticoside and madecassoside have been preliminarily constructed, emphasizing the key enzymatic steps involved (Fig. 6E). The pathway demonstrates how 2,3-oxidosqualene is initially converted to α-amyrin by the enzyme *CaDDS*. Subsequent modifications involve multiple CYP enzymes and UGTs, which catalyze the formation of various intermediates, such as ursolic acid and asiatic acid, before ultimately leading to the biosynthesis of asiaticoside and madecassoside. The precise role of different CYPs, including *CaCYP716A83* and *CaCYP716C11*, and UGTs, such as *CaUGT1* and *CaUGT73AH1*, is emphasized in the context of glycosylation and hydroxylation reactions essential for triterpenoid biosynthesis (Fig. 6E).

## Discussion

In this study, we present the first complete T2T genome assembly for *C. asiatica*, following the carrot (*D. carota*) as the only other Apiaceae family member to have such an assembly [35]. Compared to the chromosome-level assembly reported by Pootakham *et al*. (BB-174) [26], our assembly exhibited superior contiguity (only 4 gaps), completeness (BUSCO score of 99%), and accuracy (QV score of 44.45). This high-quality genome provides a valuable resource for investigating the species-specific biosynthesis of pentacyclic triterpenoid saponins in Apiaceae.

Comparative genomics analysis provides significant insights into species differentiation, gene family dynamics, and WGD events, etc. [36, 37]. *C. asiatica*, a prominent Apiaceae species characterized by its pentacyclic triterpenes (asiaticoside and madecassoside), serves as a key model for elucidating the origin of genes involved in the biosynthesis pathway of its unique chemical components. Comparative genomics analysis revealed that *C. asiatica* is an early-diverging lineage within the Apiaceae family, and diverged from ancestral Apiaceae species ~53.1–66.22 million years ago. *C. asiatica* has experienced a shared WGD event (Apiaceae-ω) within the Apiales subsequent to the ancient γ-triplication, potentially shaping its unique gene content and evolutionary trajectory, which is in line with previous research [36, 37]. In contrast, other Apiaceae species have two recent WGDs (Apiaceae-α and Apiaceae-ω) [38, 39]. Specifically, *C. asiatica* has fewer number of *CYP* and *UGT* genes, which also suggests that it may not have undergone the recent WGD events shared by other Apiaceae plants. This discrepancy in WGD may reflect lineage-specific evolutionary pressures or ecological adaptations that have uniquely influenced the genomic landscape of *C. asiatica*. Moreover, the genome of *C. asiatica* contains 16 906 gene families, including 188 gene families that are unique to this species. Two of the known genes belonging to these unique gene families, *UGT73AD1* and *UGT1*, are involved in the biosynthesis of asiaticoside and madecassoside, key compounds in *C. asiatica*.

Furthermore, 3D chromatin structure analysis highlighted the critical role of A/B compartment organization in regulating the expression differences of WGD genes in *C. asiatica*. The observed enrichment of WGD genes within the A compartments, which are characterized by higher gene density and transcriptional activity, underscores the functional importance of these genomic regions. Moreover, the differential expression patterns of WGD gene pairs, especially those located in different A/B compartments, suggest that chromatin architecture may drive the functional divergence of duplicated genes during the diploidization process. Particularly, we discovered that two WGD genes (*Cas07G002524* and *Cas03G000219*) belong to the UGT73 family in *C. asiatica*, which are associated with the biosynthesis of asiaticoside, and are in different chromatin states, leading to alterations in gene expression. This finding aligns with existing literature on plant chromatin structure but adds new insights by demonstrating that changes in compartmental status can significantly impact the regulation of key metabolic pathways [40]. Nevertheless, the current study has limitations, including the resolution of Hi-C data, which may restrict the identification of finer chromatin structures. Future studies employing higher resolution Hi-C data or complementary chromatin conformation capture techniques could provide deeper insights into the relationship between chromatin architecture (loop) and gene regulation in *C. asiatica*.

The diversity of triterpenoid saponins in *C. asiatica* likely stems from genomic variations and specific expression, particularly in the 2,3-oxidosqualene cyclase genes that form the triterpenoid skeleton, and subsequent modifications by CYPs and UGTs [41, 42]. Our gene family analysis identified 11 OSCs, 160 CYPs, and 117 UGTs. Moreover, in the *OSC* gene family, *CasiOSC03* has been identified as encoding DDS, which catalyzes the conversion of 2,3-oxidosqualene into the asiaticoside backbone α-amyrin [43]. This gene shows high expression across different Apiaceae species. However, a single functional α-amyrin synthase has not yet been confirmed, suggesting a prolonged evolutionary development of this enzyme. Key gene families, including CYP716, CYP714, and UGT73, are crucial for the biosynthesis of asiaticoside and madecassoside in *C. asiatica*. The expansion of these families is primarily driven by both tandem and whole-genome duplications. Although *C. asiatica* has not experienced the recent WGD event that is widespread among other Apiaceae species, resulting in a general reduction in the two key gene families (CYP and UGT) crucial for triterpene biosynthesis, our research has revealed an unexpectedly rich abundance of highly expressed genes within subfamilies like CYP714, CYP716, and UGT73 in the leaf tissue, which are the known gene subfamilies for asiaticoside and madecassoside production. This observation indicated that gene family efficacy may rely on the number of highly expressed genes in the key subfamilies. Moreover, the key genes responsible for asiaticoside and madecassoside biosynthesis, including *CasiOSC03*, *CYP714E19*, *CYP716C11*, and *UGT73AD1*, exhibit a lack of collinearity with their counterparts in other Apiaceae species, suggesting that the special nonconserved gene organizations may be associated with the biosynthesis of these compounds. Although the UGT73 family genes of *C. asiatica* are not located within the *OSC* gene clusters, it is particularly striking that these *UGT73* genes have experienced distinctive and nonconservative expansion, forming tandem gene clusters. These clusters were strategically positioned within the same TAD, suggesting that *C. asiatica* may enhance the biosynthesis of asiaticoside and madecassoside by forming gene clusters and capitalizing on the 3D genome architecture to facilitate coordinated gene expression. This pronounced upregulation of functional genes in *C. asiatica* is consistent with observations in other plant species, such as *Kingdonia uniflora* [44], where gene family contraction is linked to adaptation to stable environments. Overall, these findings demonstrate how the interplay of gene family expansion, contraction, and ancient WGDs has shaped the biosynthetic landscape of triterpenoid saponins in *C. asiatica*.

MeJA treatment, which increases triterpenoid content by 1.4-fold [45–47], was shown to significantly upregulate genes associated with triterpenoid biosynthesis at 24 h. The identification of specific gene clusters and coexpression modules, particularly the 'skyblue' module enriched in terpenoid biosynthesis pathways, highlights the regulatory complexity involved in asiaticoside and madecassoside biosynthesis. Previous studies have reported the influence of overexpressed squalene synthase (SQS) and the TF *TSAR2* on triterpenoid content [15]. Notably, the genes *Cas01G000594* and *Cas04G00117/118* are homologous to *AtbHLH92* and *AtbHLH14*, respectively, while *Cas02G000621* is homologous to *AtTIFY6B*. These bHLH and TIFY TFs are involved in stress responses, MeJA signaling, and secondary metabolism [48, 49]. However, the study of MeJA treatment is limited by the focus on transcriptional data, which may not fully capture the complexity of post-transcriptional and post-translational modifications involved in triterpenoid biosynthesis. Future studies will explore these aspects to further elucidate the regulatory networks.

In summary, based on a comprehensive genomic and transcriptomic analysis of *C. asiatica*, this study provides a foundational framework for understanding the molecular basis of triterpenoid saponin biosynthesis and its regulation. Our findings reveal the high expression of a special subfamily, nonconserved gene organization, and coexpression networks that collectively contribute to the biosynthetic pathways, opening avenues for targeted genetic manipulation to enhance saponin production. These insights not only deepen our knowledge of the regulatory complexity within the *C. asiatica* genome but also offer a valuable resource for future studies aimed at metabolic engineering and drug development.

## Materials and methods

### Sample preparation, genomic library construction, and genome sequencing

Fresh and healthy leaves, roots, and stems were harvested from 1-month-old *C. asiatica* plants, immediately frozen in liquid nitrogen, and stored at −80°C. The quality and quantity of the extracted genomic DNA were assessed via a NanoDrop One spectrophotometer and a Qubit 3.0 fluorometer, respectively. Eligible DNA was then used to construct an NGS library, which was sequenced on the DNBSEQ-T7 platform with 350-bp paired-end reads. High-fidelity (CCS HiFi) reads were obtained through single-molecule sequencing on the PacBio Sequel IIe platform. Additionally, Hi-C sequencing libraries were prepared, amplified by PCR (12–14 cycles), and sequenced on the BGI platform following standard Hi-C library preparation protocols [50].

### Genome assembly and quality assessment

To obtain clean reads, Trimmomatic (v0.39) [51] was used to remove adapters and filter out low-quality sequences. Genomic ploidy was assessed using Smudgeplot (v0.2.5), while genome size and heterozygosity were estimated with GCE (v1.0.2) based on k-mer frequency (k = 17) [52, 53]. PacBio HiFi reads were *de novo* assembled into contigs via hifiasm (v0.16.1) [54] and visualized with Bandage2 [55]. Hi-C technology was employed to anchor contigs to chromosomes, starting with the removal of adapters and low-quality reads using Trimmomatic (v0.39) with specific parameters (LEADING:20, TRAILING:20, SLIDINGWINDOW:4:20, MINLEN:50). Chromosome-level scaffolds were then constructed using YaHS (v1.1) [56] with Hi-C data. Juicer (v1.6) and Juicerbox (v1.11.08) [57] were utilized to generate interaction heatmaps, allowing for the identification and manual correction of potential scaffolding errors. Gaps in the scaffolds were closed using quarTeT (v1.2.1) [58], which also predicted telomeres and centromeres of the pseudochromosomes. StainedGlass (v0.9) [59] was used to generate pairwise sequence identity heatmaps for centromeres. The final Hi-C interaction heatmap was generated with plotHicGenome (v0.1.0). The Barrnap v0.9 (https://github.com/tseemann/barrnap) was used to predict the location of rDNA in the genome and validated with two published 45S rDNA sequences: KM036297.1 (*Panax quinquefolius*) and KM036295.1 (*Panax ginseng*).

Genome assembly statistics, including genome size, N50, and gap count, were calculated using QUAST (v5.0.2) [60]. Genome completeness was evaluated via BUSCO (v5.4.7) [61] with the embryophyta_odb10 database, while Merqury (v1.3) [62] was applied to assess completeness and base accuracy using NGS reads. Additionally, the LAI was calculated with LTR_retriever (v2.8) [63]. Structural variation analysis between the BB-174 and wild-type *C. asiatica* genomes was conducted using SyRI (v1.6).

### Genome annotation

Repetitive sequences in the *C. asiatica* genome were identified and classified using EDTA (v2.1.0) [64], followed by masking with RepeatMasker (v4.1.2) (http://www.repeatmasker.org/RepeatMasker/). For noncoding RNA annotation, snRNAs and miRNAs were identified with Infernal (v1.1.4), tRNAs with tRNAscan-SE (v2.0.9) [65], and rRNAs with barrnap (v0.9) (https://github.com/tseemann/barrnap). Protein-coding genes were annotated through a combination of *ab initio*, homology-based, and transcript-based methods. *Ab initio* predictions were performed using AUGUSTUS (v3.4.0) [66], GlimmerHMM (v3.0.4) [67], geneid (v1.4.5), SNAP (v2006-07-28), GeneMark (v4.68) [68], Genscan (v1.0,), and BRAKER (v2.1.6) [69]. Homology-based predictions were conducted by mapping protein sequences from reference genomes (*Arabidopsis thaliana*, *D. carota*, *P. notoginseng*, *P. ginseng*, *C. sativum*, *A. sinensis*) using GeMoMA (v1.8) [70].

Transcript-based predictions involved mapping clean data to the genome with HISAT2 (v2.2.1) [71] and assembling transcripts using StringTie (v2.2.1) and Trinity (v2.13.2) [72]. The coding regions of transcripts were predicted by TransDecoder (v5.5.0). Subsequently, the assembled transcripts were used for gene structure prediction with PASA (v2.5.1) [73]. Gene structures derived from different methods were integrated using EVidenceModeler (v1.1.1) and the weights score for each type of evidence were set as follows: Homology-set = 2, transcripts-set = 10, and *Ab initio* set = 1. Finally, the gene models were updated by PASA (v2.5.1) [73]. Genome annotation completeness was assessed using BUSCO (v5.4.7) [61]. Functional annotation of predicted protein-coding genes was achieved through homology searches against databases such as NR, KEGG, KOG, TrEMBL, and SwissProt using DIAMOND (v2.1.9) [74]. PfamScan (v1.6) aligned protein sequences to the Pfam database (v36.0), and Gene Ontology (GO) terms and KEGG pathways were annotated via InterProScan (v5.66–98.0) and KofamScan (v1.3.0) [75, 76], respectively.

### Comparative genomics analysis

To infer orthologs and orthogroups, nonredundant protein sequences from 17 species were analyzed using Orthofinder (v2.4) [29]. A total of 1014 orthogroups, with a minimum of 76.5% of the species having single-copy genes, were subjected to alignment via MAFFT (v7.205) [77] and Gblocks (v0.91b) [78] to eliminate poorly aligned or highly divergent regions. The optimal model (JTT + F + I + G4) was determined via IQ-TREE's ModelFinder (v1.6.11) [79] and used to build the tree with a 1000-bootstrap maximum likelihood method [80]. Divergence times were estimated using MCMCTREE [81] in PAML (v4.9i) [82], with corrections based on TimeTree (http://www.timetree.org/) data for key species pairs. The fossil divergence times are as follows: 179.9–205.0 million years for *Amborella trichopoda* versus *V. vinifera*, 111.4–123.9 million years for *V. vinifera* versus *D. carota*, 76.0–97.4 million years for *D. carota* versus *E. breviscapus*, 54.3–69.0 million years for *D. carota* versus *E. senticosus*, 28.405–31.395 million years for *P. notoginseng* versus *P. vietnamensis*, 35.15–38.85 million years for *D. carota* versus *H. sosnowskyi*, 1.634–1.806 million years for *O. javanica* versus *O. sinensis*, and 5.4–40.2 million years for *O. javanica* versus *C. sativum*. Orthogroups with a large size variance among species were identified by CAFE (v4.2) [83] with a significance threshold set at family-wide *P*-values <.05. Additionally, orthogroups with accelerated rates of evolution were further determined with branch-specific Viterbi *P*-values <.05. Positive selection among *C. asiatica* and related species was examined using branch-site models in PAML [82]. Collinearity analysis was conducted with MCScanX [84] and visualized using JCVI (v0.9.13) (https://github.com/tanghaibao/jcvi). Meanwhile, WGDs were detected by calculating Ks values with WGDI (v0.6.0) [85] and substitution-rate-adjusted mixed paralog–ortholog plot for *C. asiatica* as produced by ksrates (v1.1.4) [86]. The LTR-RT insertion times were estimated by LTR_retriever (v2.8) [63] (for candidate LTR-RT identification) and EMBOSS (v6.6.0) (for distance (K) of LTR calculation), with a molecular clock rate of 7 × 10^−9^ substitutions per site per year. Finally, gene duplication was classified using the Duplicate_gene_classifier in the MCScanX package [84], and GO and KEGG enrichment analyses were conducted with clusterProfile (v3.4.4) [87].

### A/B compartment and TAD identification

Hi-C data normalization was performed using Hi-C-Pro (v2.10.0) [88] and the resolution was >5 kb. Subsequently, principal component analysis (PCA) was applied to the 100 kb normalized matrix using HiTC (v1.24.0), with PC1 values designating A and B compartments as positive and negative, respectively. Furthermore, TADs were identified using HiCexplorer (v3.7) [89] on the 5 kb normalized matrix.

### *OSCs*, *CYP450*, and *UGT* gene family identification

Known protein sequences for the gene families (OSC, CYP450, and UGT) were retrieved from the NCBI database. Candidate genes were identified using BLASTP [90] with an E-value threshold of <1e–5. Validation of these candidates was further performed using HMMER3.0 [91], with parameters set to (−E 0.01 and −incdomE 0.00001), against specific domain models, including the squalene–hopene cyclase N-terminal domain (PF13249), C-terminal domain (PF13243), P450 domain (PF00067), and UDP-glucoronosyl/UDP-glucosyl transferase (PF00201). Phylogenetic relationships were then analyzed using MEGA X [92] software, constructing a Neighbor-Joining (NJ) tree with bootstrap support based on 1000 replicates.

### Metabolite analysis

The frozen leaves of *C. asiatica* (50 mg) were vacuum-frozen, and the homogenate was resuspended in prechilled 70% methanol by vortexing six times (intervals of 30 min). Then the samples were centrifuged at 15 000 g for 3 min and filtered for the UPLC-MS/MS analysis. The samples were injected into an Agilent SB-C18 (2.1 × 100 mm, 1.8 μm) with a 14-min linear gradient at a 0.35 ml/min flow rate. The eluents used were eluent A (0.1% formic acid water) and eluent B (0.1% formic acid-acetonitrile). The solvent gradient was set as follows: 5%–95% B, 9 min, 95% B, 10–11.0 min, 95%–5% B, 14.0 min. The data files generated were processed using Analyst v1.6.3(https://sciex.com/products/software/analyst-software).

### Transcriptome analysis of MeJA treatment

The 2-month-old *C. asiatica* plants were cultivated in a growth chamber at a temperature of 22°C, with a 16-h light and 8-h dark photoperiod. Foliar sprays of three of these plants were treated with 200 ml of 200 μmol MeJA solution. Leaves and stems were collected at seven time points (0, 3, 6, 9, 12, 18, and 24 h), with three biological replicates per time point. The samples were immediately frozen and stored at −80°C. Total RNA extraction and cDNA synthesis were performed following the protocol. mRNA libraries were constructed and sequenced using Illumina NovaSeq with 250-300-bp paired-end reads. Clean reads were mapped to the reference genome using HISAT2 v2.2.171 [71], and gene expression levels were quantified with Stringtie v2.1.4 [93]. A comprehensive WGCNA was performed using the r package (wgcna), and the correlation network was visualized with Cytoscape [94].

### Functional characterization of *OSC* genes in *Saccharomyces cerevisiae*

The cDNA encoded in the ORFs of *CaOSC1 ~ CaOSC11* was ligated to the under GAL1 promoter of the *p*YES2 expression vector via homologous recombination to generate *p*YES2-*CaOSCs*, respectively. The lithium-acetate Method was used to transform these plasmids into the mutant strain in GIL77 [95]. The empty vector was also transformed into strains as a control. The transformed yeast cells were selected for the uracil prototype and were grown at 30°C for 2 days. Briefly, the yeast strains were cultivated in media supplemented with glucose (2%) for 3 days, and then transferred to fresh media containing galactose (2%) and incubated at 30°C for 48 h.The induced cells were then transferred to a resting medium containing phosphate buffer (pH 7.0) for 12 h at 30°C, and then cell disrupted with 20% KOH/50% EtOH at 98°C for 5 min. The sample was extracted from petroleum ether three times and then underwent trimethylsilyl (TMS) derivatization. Gas chromatography–mass spectrometry (GC–MS) was used to analyze derivatized samples, based on their retention times and mass spectra to separate and identify the compounds [31].

## Supplementary Material

Web_Material_uhaf037

## Data Availability

The raw genome sequencing and transcriptome data reported in this study are accessible through NCBI under the accession number PRJNA1121499 (https://www.ncbi.nlm.nih.gov/Traces/study/?acc=SRP512688&o=acc_s%3Aa). The genome assembly and annotation data can be found in the Figshare database at https://doi.org/10.6084/m9.figshare.28022099.v1 and the National Genomics Data Center (NGDC) at https://ngdc.cncb.ac.cn/gsa/, with the BioProject identifier PRJCA033632.
