# Supplementary material for: Telomere-to-telomere genome assembly and 3D chromatin architecture of Centella asiatica insight into evolution and genetic basis of triterpenoid saponin biosynthesis
Source: Hortic Res. 2025 Feb 7;12(5):uhaf037. doi: 10.1093/hr/uhaf037 (PMC11997435; doi:10.1093/hr/uhaf037)
Supplement: Web_Material_uhaf037 [file web_material_uhaf037.zip › supplementary figure-2025-0113.pdf]

Supplemental information

Supplementary Figures

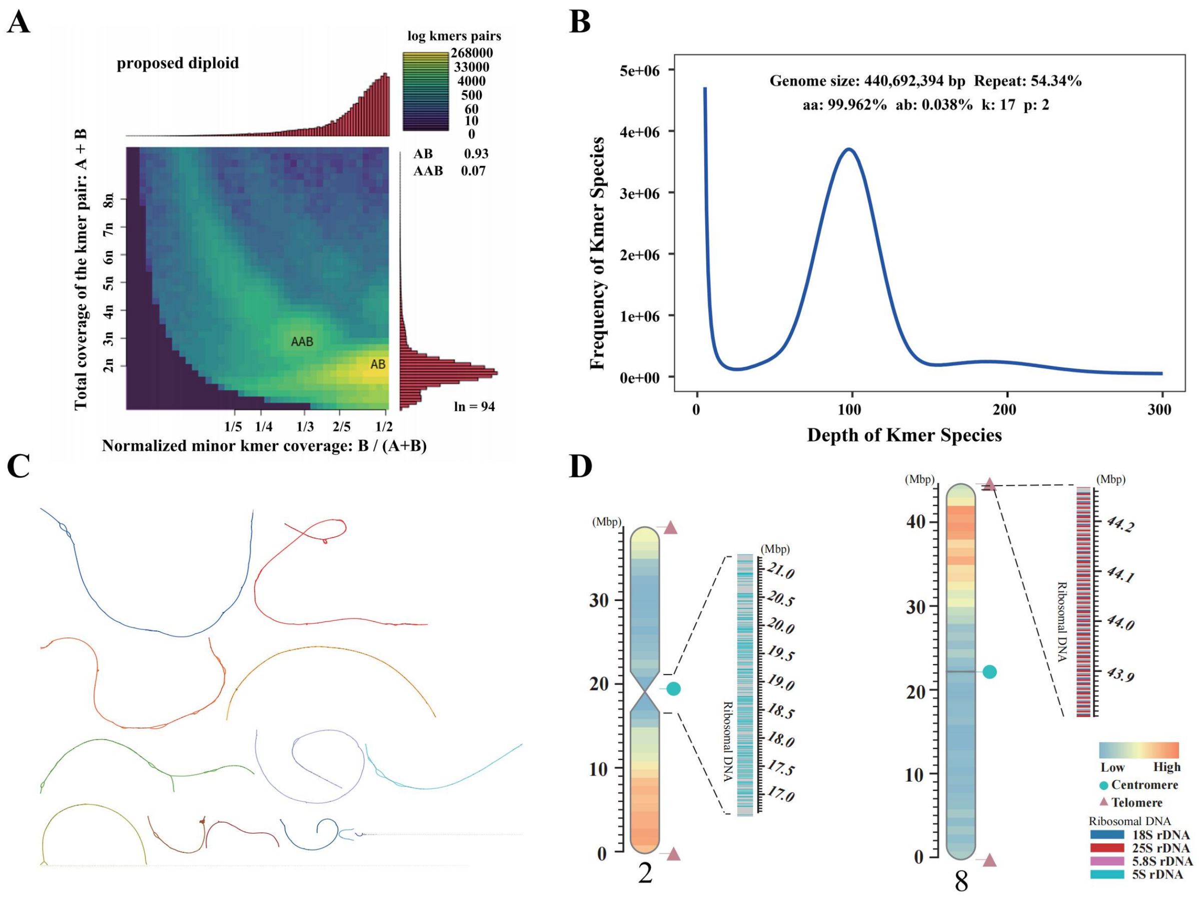

Figure S1. Genome survey and assembly evaluation. Smudgeplot analysis based on k-mers (A) and k-mer 17 distribution of *C. asiatica* the genome (B). The horizontal axis of the heatmap in (A) represents the relative coverage (CovB / (CovA + CovB)), while the vertical axis indicates the total coverage (CovA + CovB). The color signifies the frequency of k-mer pairs. Each haplotype structure is depicted as a "smudge" on the plot, with the intensity of the smudge reflecting the frequency of that haplotype structure within the genome. The result shows evidence of diploidy of *C. asiatica* with the frequency of the AB structure is the highest. B. K-mer frequency distributions with different depth. (C) Whole genome assembly visualization with Bandage2 showing the complete assemblies. (D) rDNA distribution in two chromosomes.

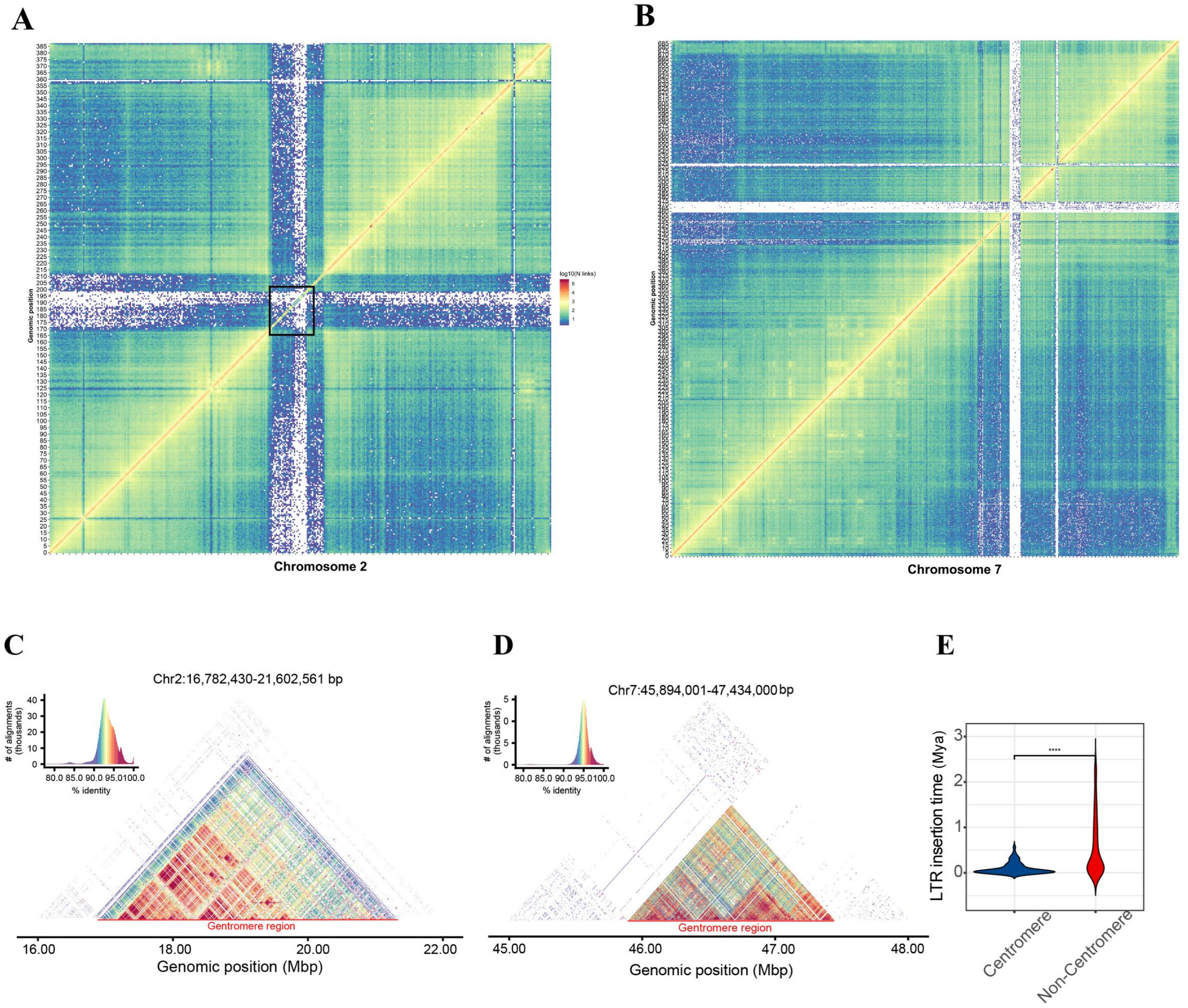

Figure S2. Validated and analysis centromere regions. Intra-chromosomal Hi-C interaction heatmaps of Chr2 (A) and Chr7 (B) in wild *C. asiatica* genome. The black boxes emphasize the centromere regions. (C) and (D) Heatmap shows pairwise sequence identity between all non-overlapping 2 kb regions surrounding the centromere. The alignment dot plot of the tandem repeats was visualized by StainedGlass, with the dot color indicating sequence identity. The red colored line marks the centromere region. (E) Compare the LTR insertion time for centromeric versus non-centromeric. We only use full length LTRs from Chr2 and Chr7 for analysis. Mya means million years ago. ****Indicates $P < 0.001$ (Wilcoxon rank-sum test).

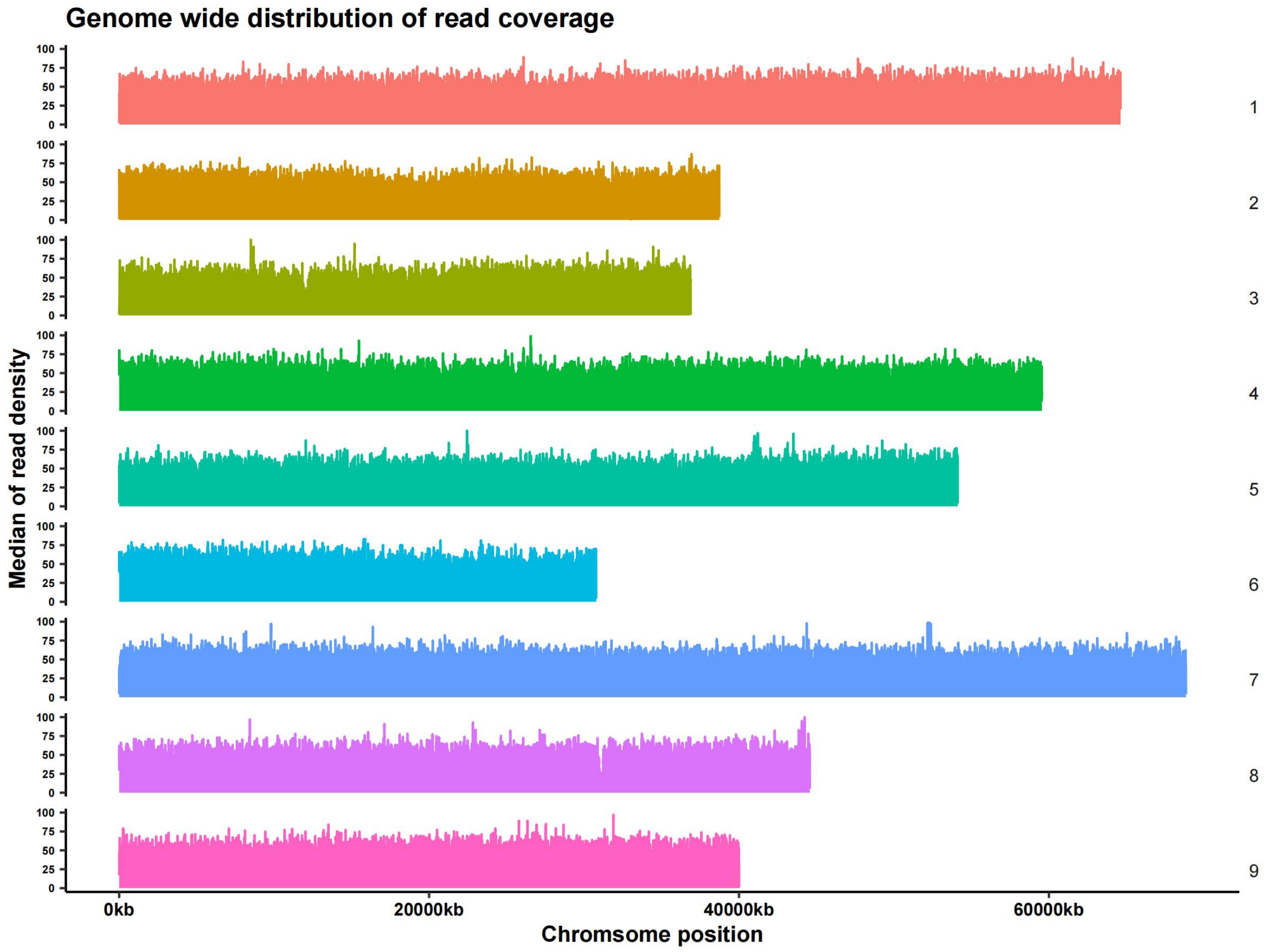

Figure S3. The read coverage depth of the wild *C. asiatica* genome. The window size for drawing is 1000bp.

A

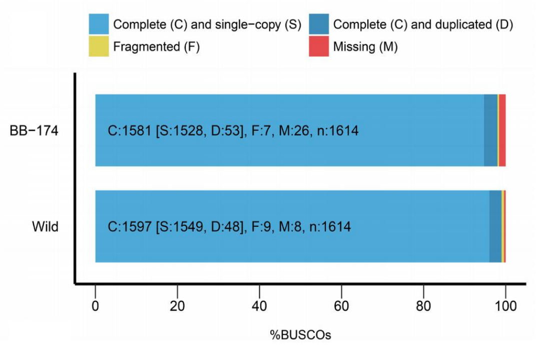

B

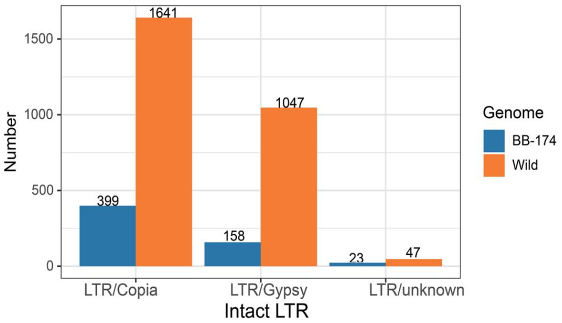

Figure S4. BUSCO and LTR analysis wild and cultivated *C. asiatica* genomes. A. BUSCO results of two genomes. C, S, D, F, M represent complete, complete and single-copy, complete and duplicated, fragmented and missing BUSCOs. **B** Intact LTR retrotransposons identified in the two genomes.

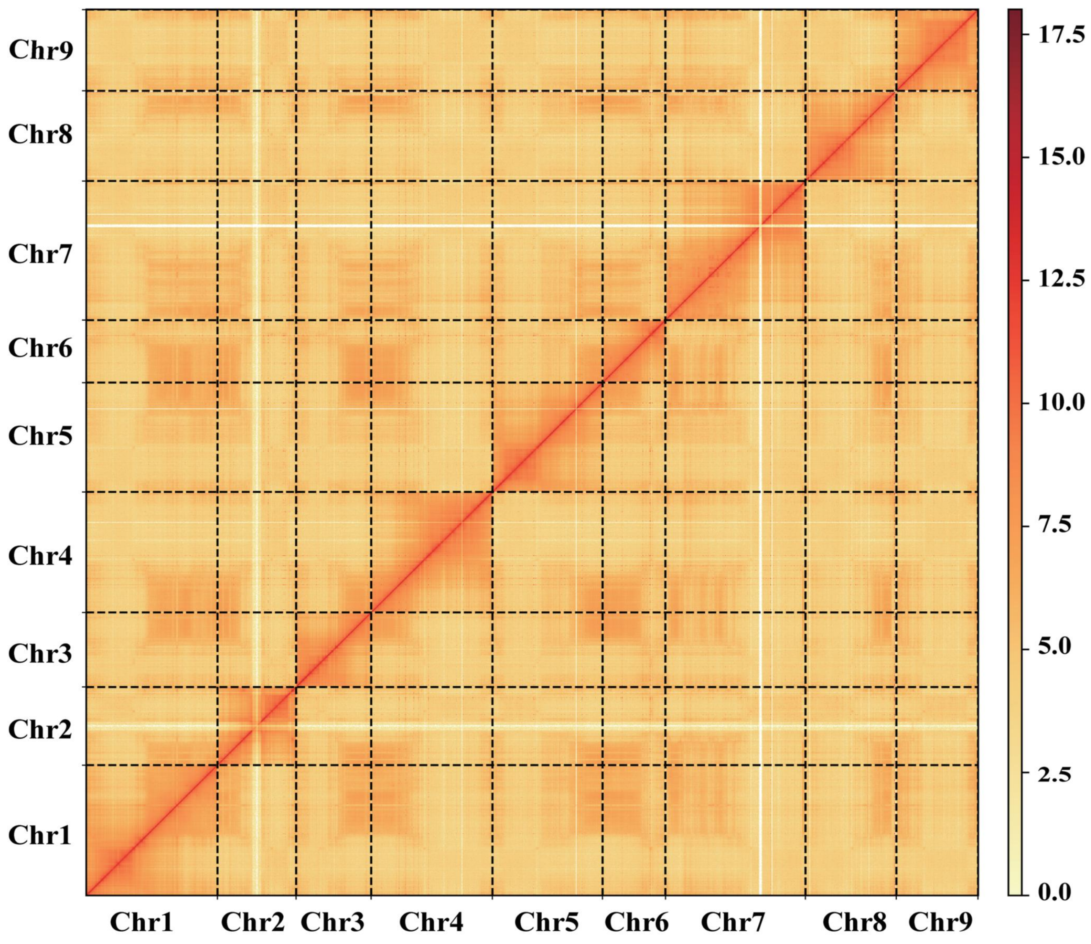

Figure S5. Hi-C interaction heatmap of 9 homoeologous chromosome groups in *C. asiatica*. The heatmaps are shown at a resolution of 100 Kb. The dark red dots indicate a high probability of interaction, while the light-yellow dots indicate a low probability of interaction.

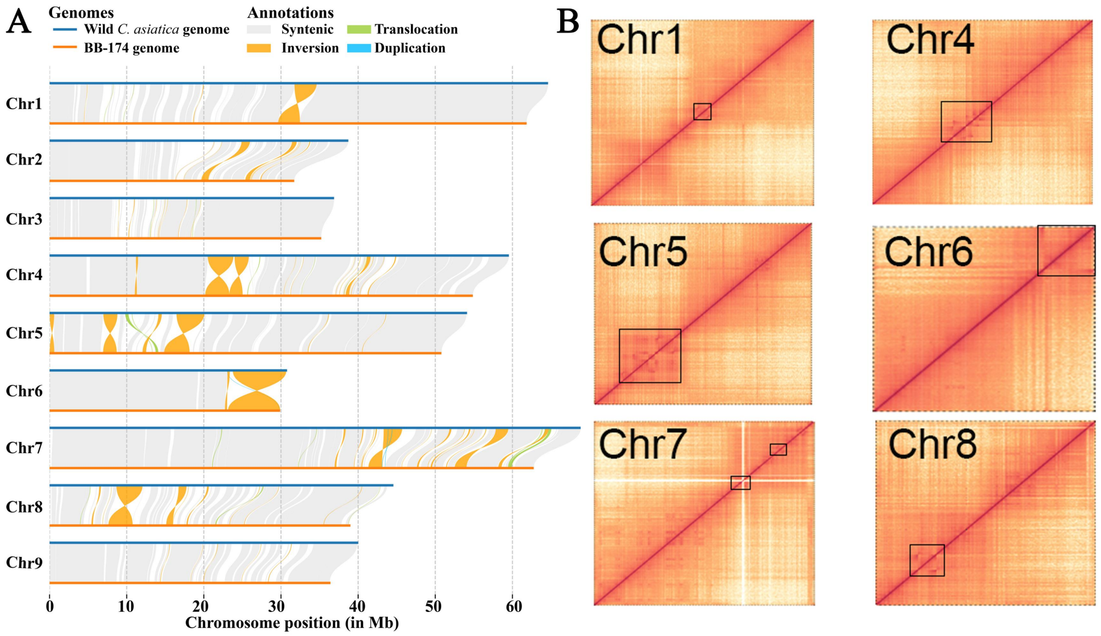

Figure S6. Structure variations between cultivate and wild *C. asiatica* genome. (A) Visualizing structural rearrangements using plotsr. Gray lines represent the syntenic regions, while orange lines represent the inversions. (B) Hi-C interaction heatmaps of BB-174 genome by mapping Hi-C data of wild *C. asiatica* against the BB-174 genome. The black boxes indicated the potential variations in BB-174.

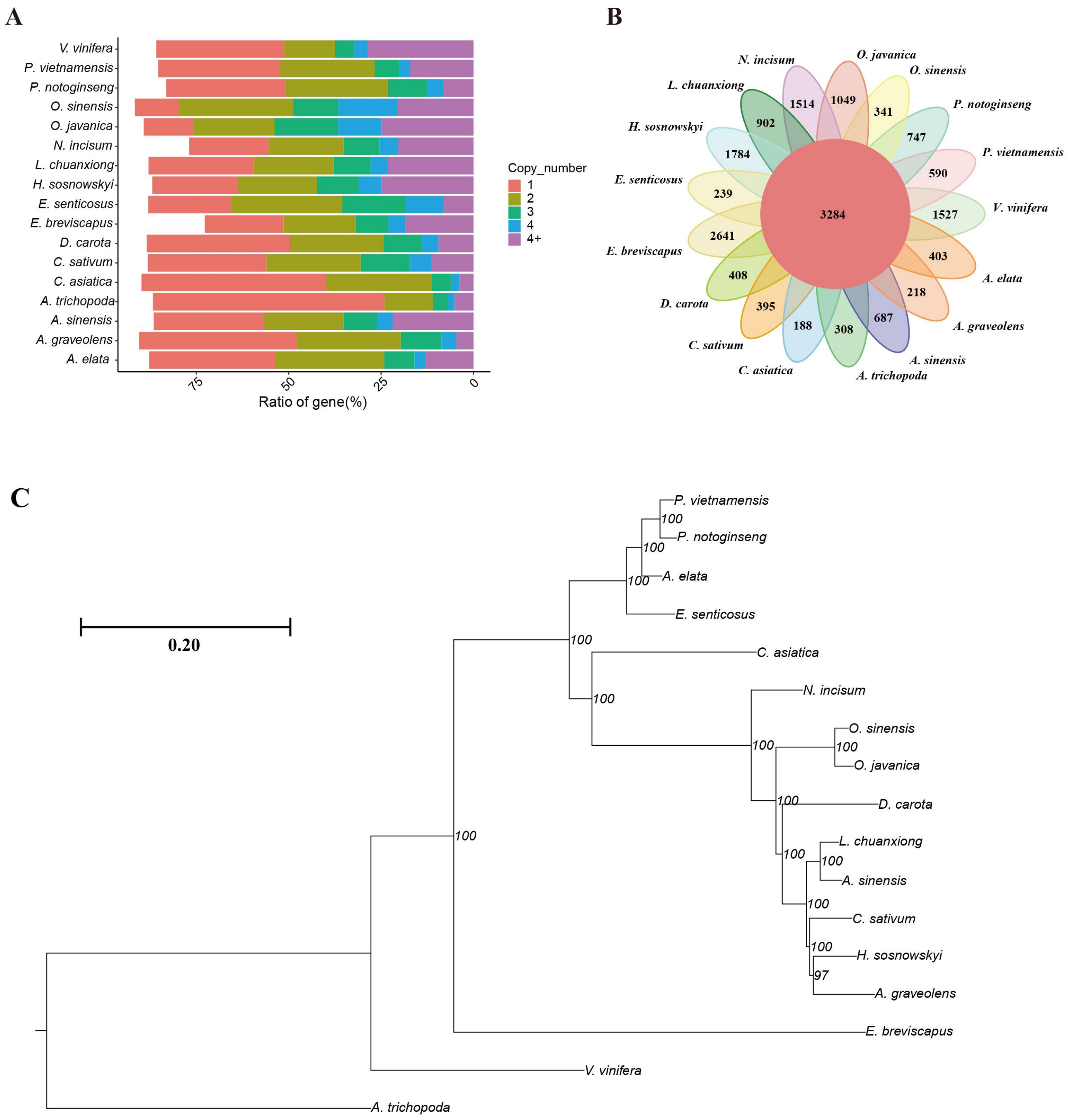

Figure S7. Orthogroups clustering and species tree construction. (A) Gene counts ratio in orthogroups distributions across 17 plants (B) Venn diagram represents the shared and unique gene families among the 17 plants. (C) Species-tree with branch length.

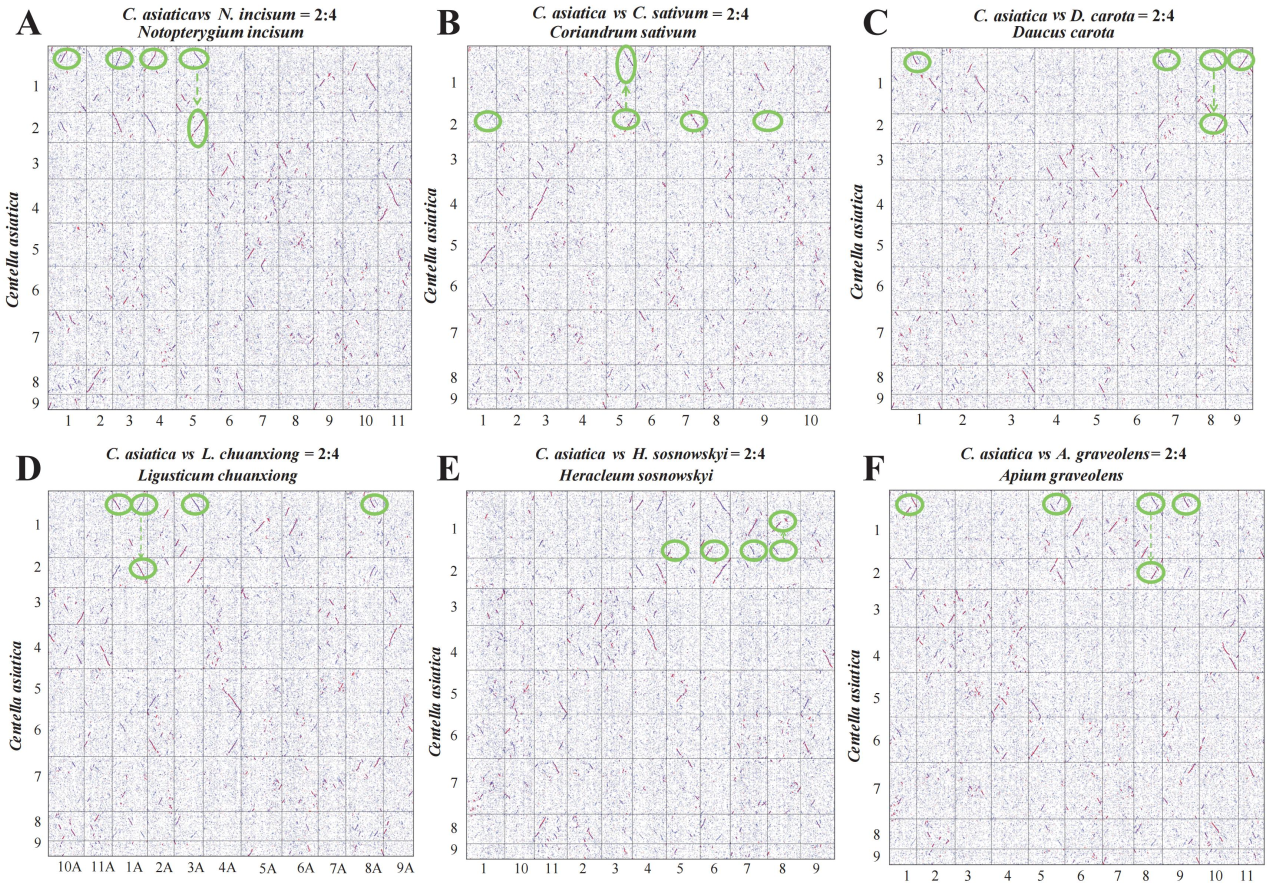

Figure S8. Dotplot analyses for *C. asiatica* and other Apiaceae species

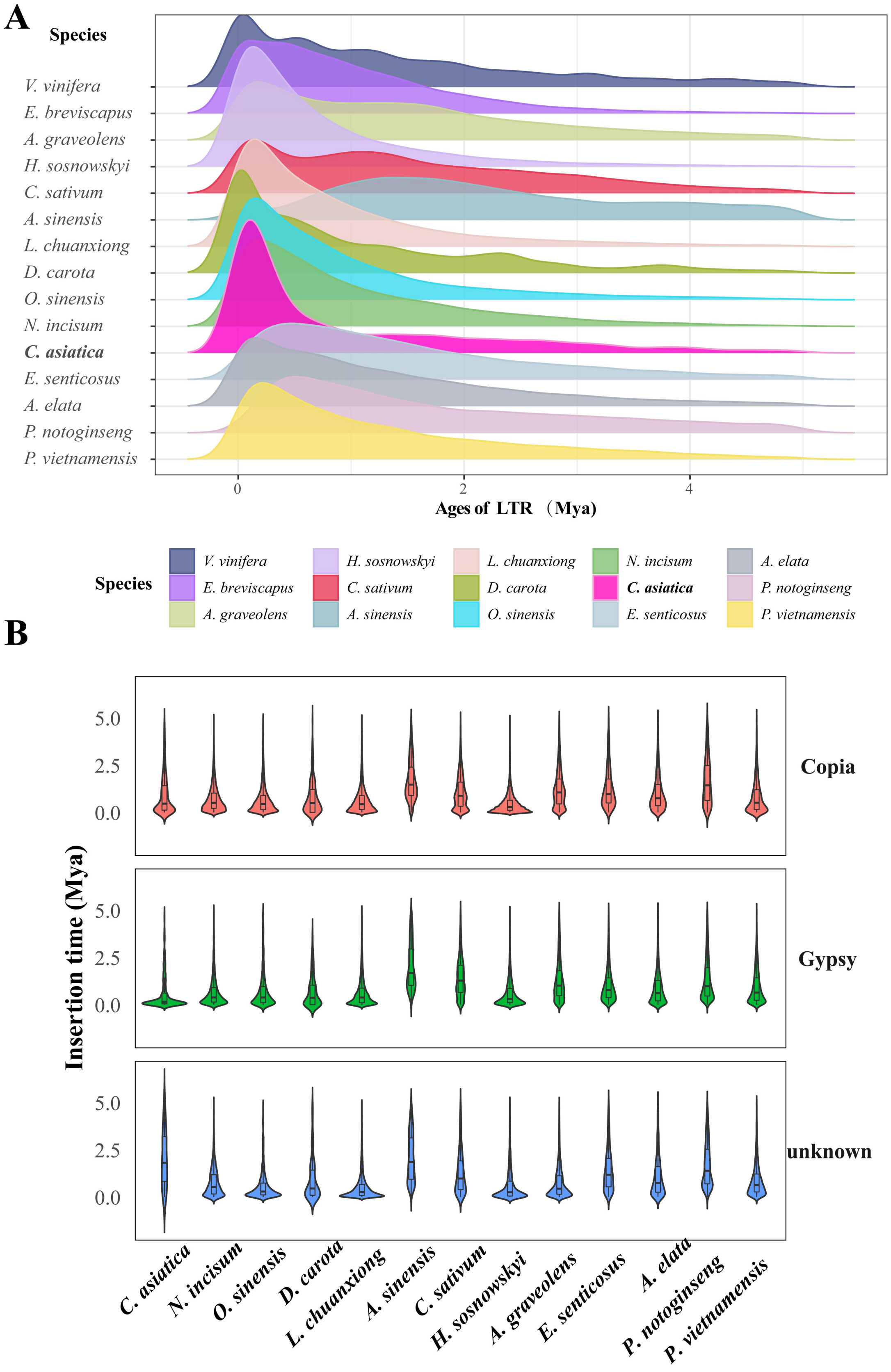

Figure S9. LTR insertion time analysis. **A** Estimated insertion time for all LTRs. **B** Estimated insertion time for Copia-LTRs, Gypsy-LTRs, and unknown type of LTRs.

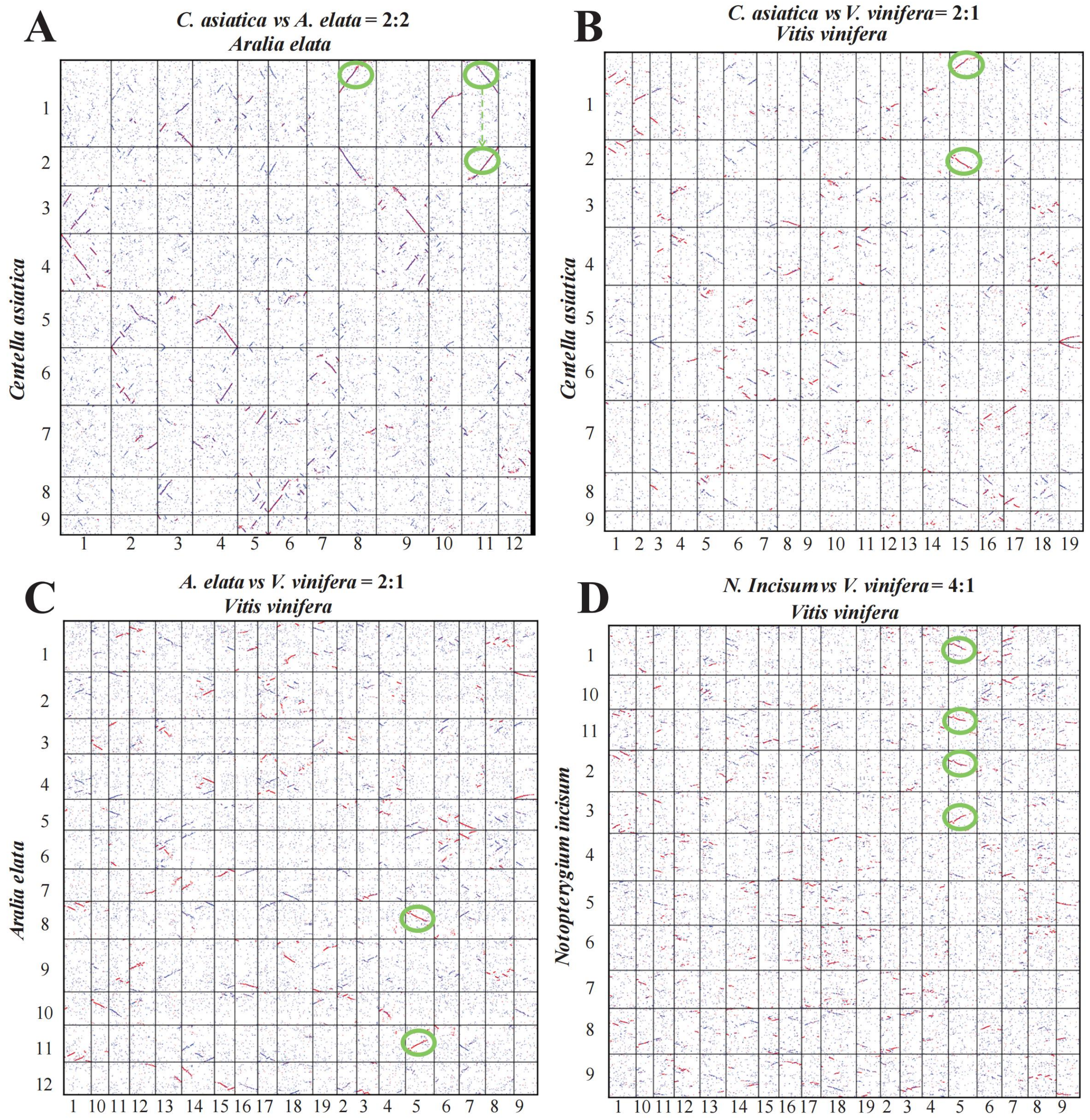

Figure S10. Orthologue dot plots between Apiaceae species and non-Apiaceae species.

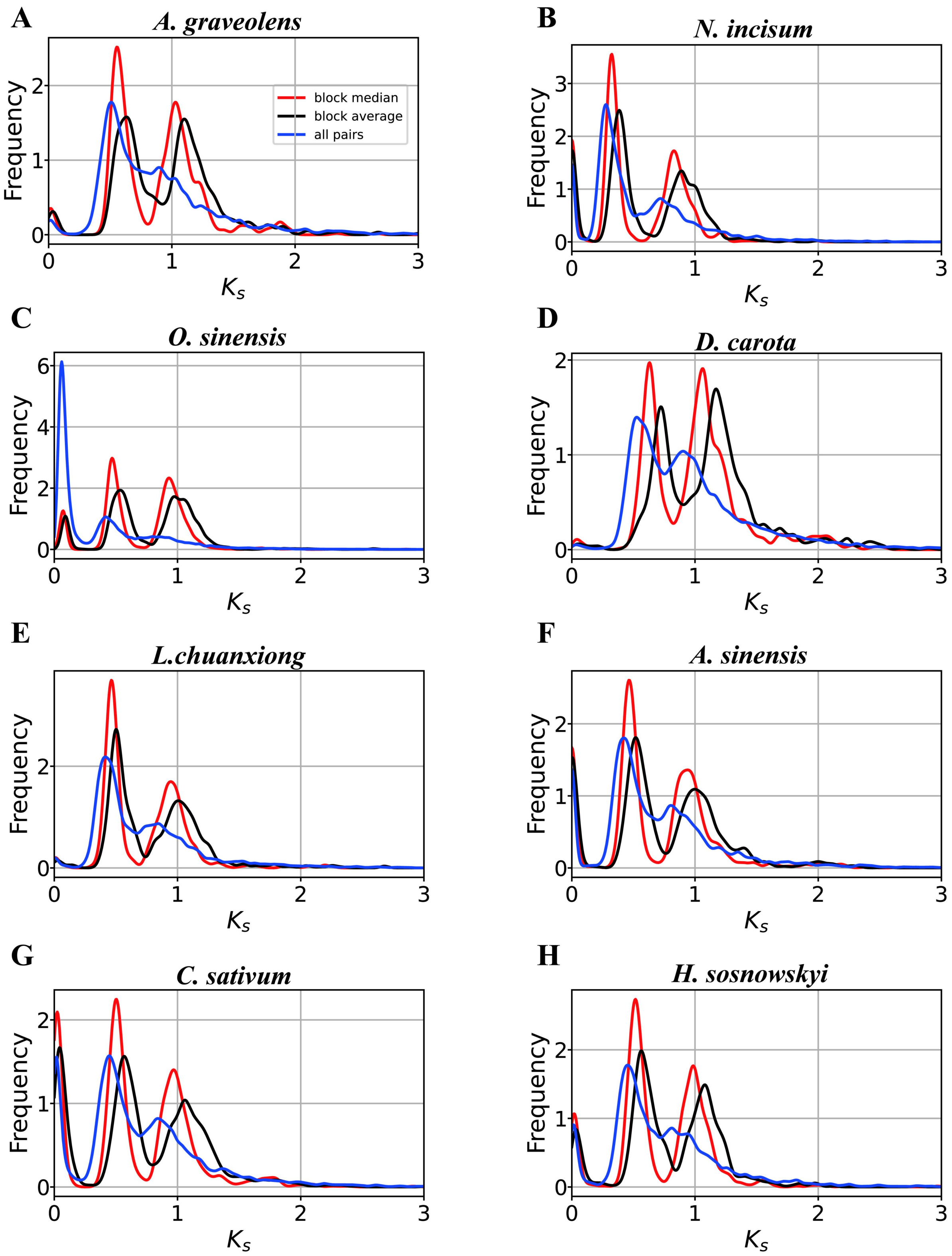

Figure S11. Ks distribution plot for species with published genome within Apiaceae Family.

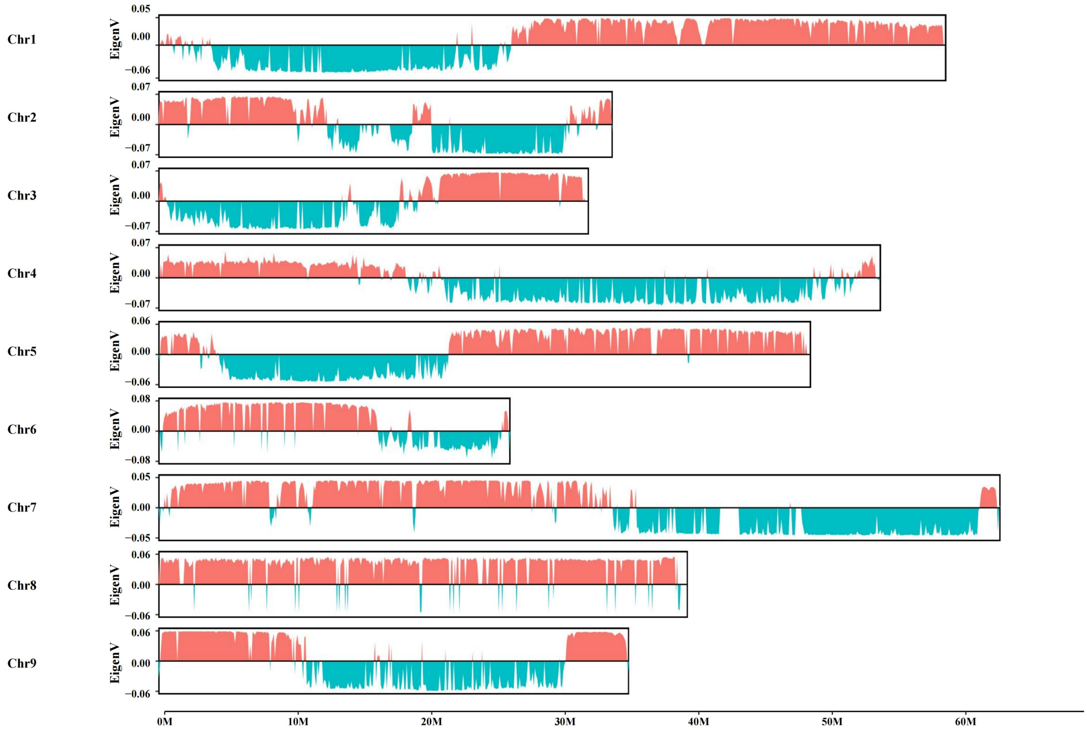

Figure S12. A/B compartments distribution in *C. asiatica* genome. Orange represents the A compartments and blue represents the B compartments.

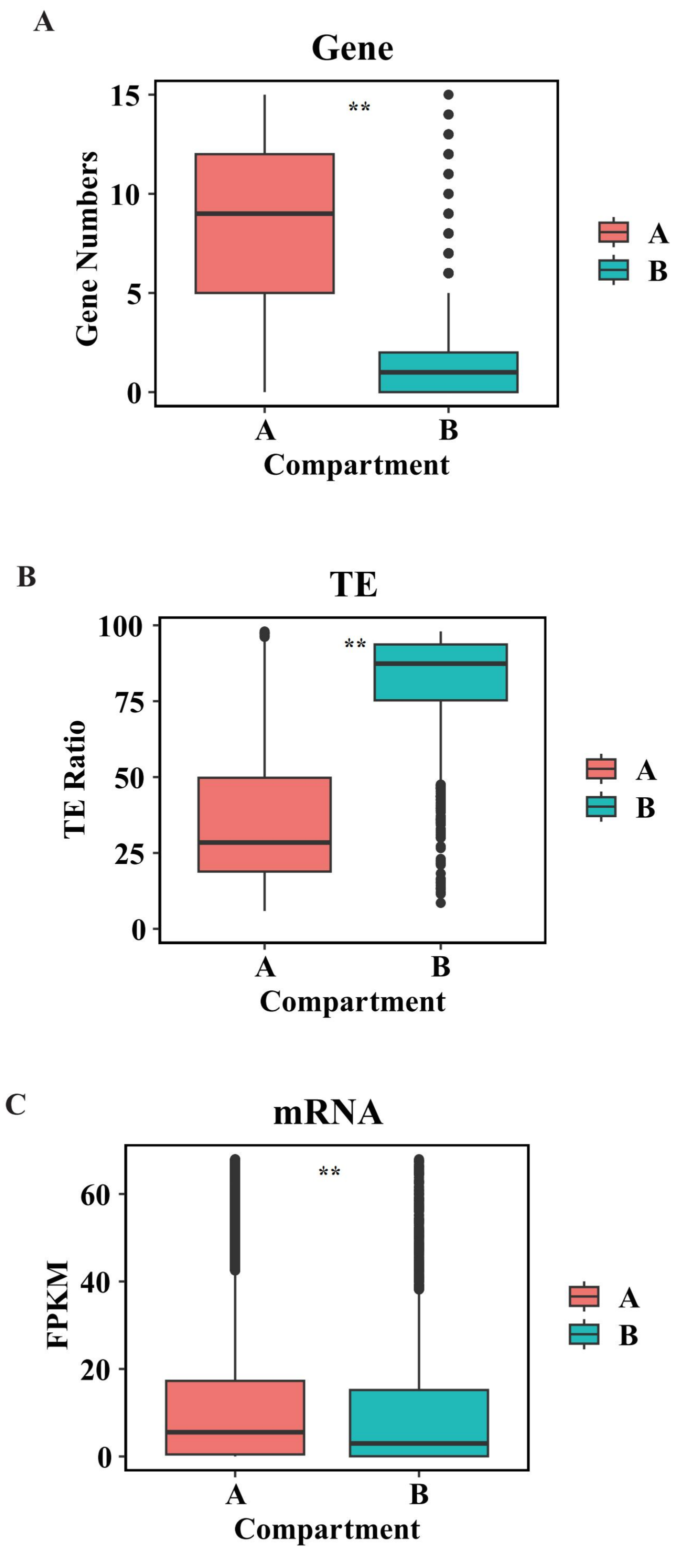

Figure S13. A/B compartment characteristics. **A-C** represents the gene density, TE ratio and gene expression level in A and B compartments. A two-sided Wilcoxon signed-rank was used for test the difference. **p < 0.001.

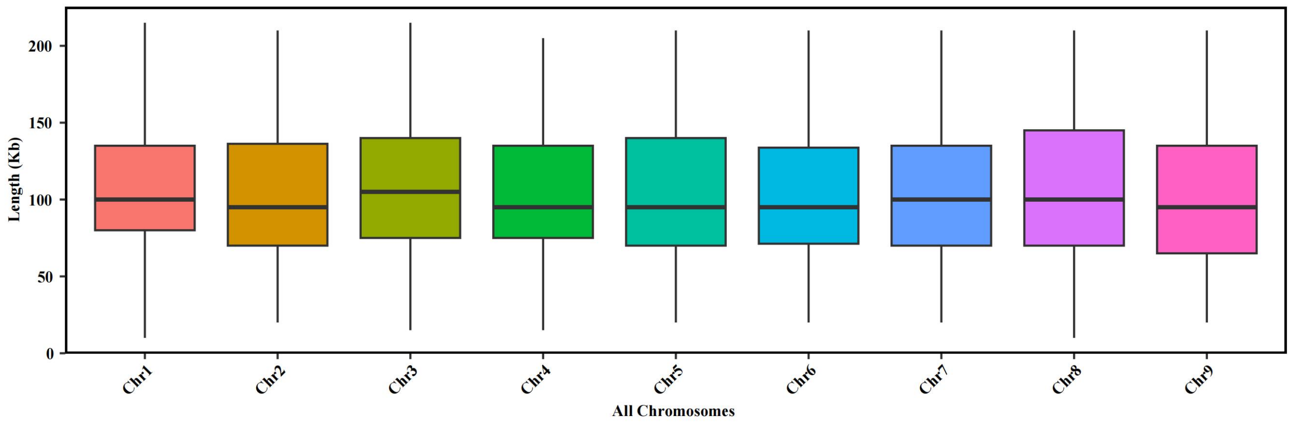

Figure S14. TAD length distribution for each chromosome.

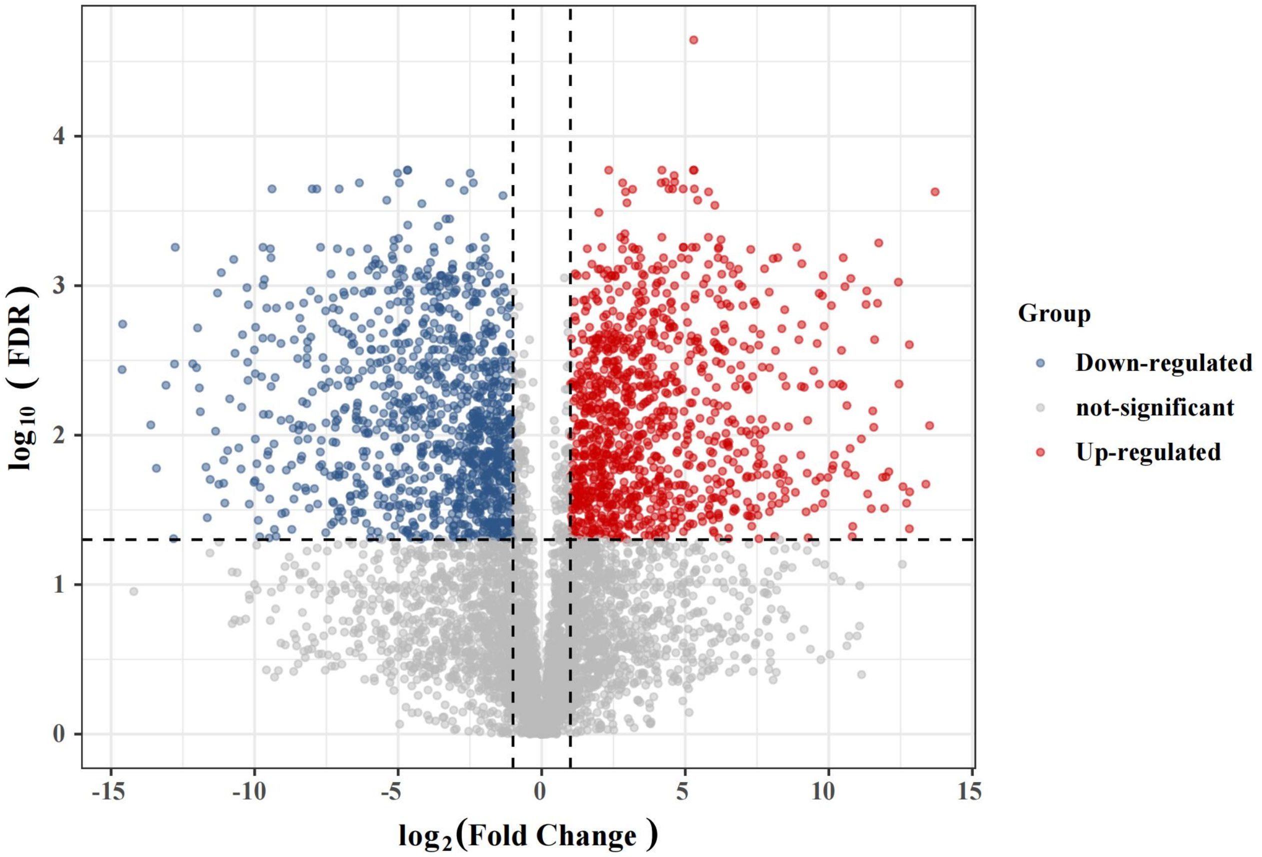

Figure S15. Volcano plots illustrating the differential gene expressions in leaf tissues between the whole-genome duplication (WGD) gene pairs.

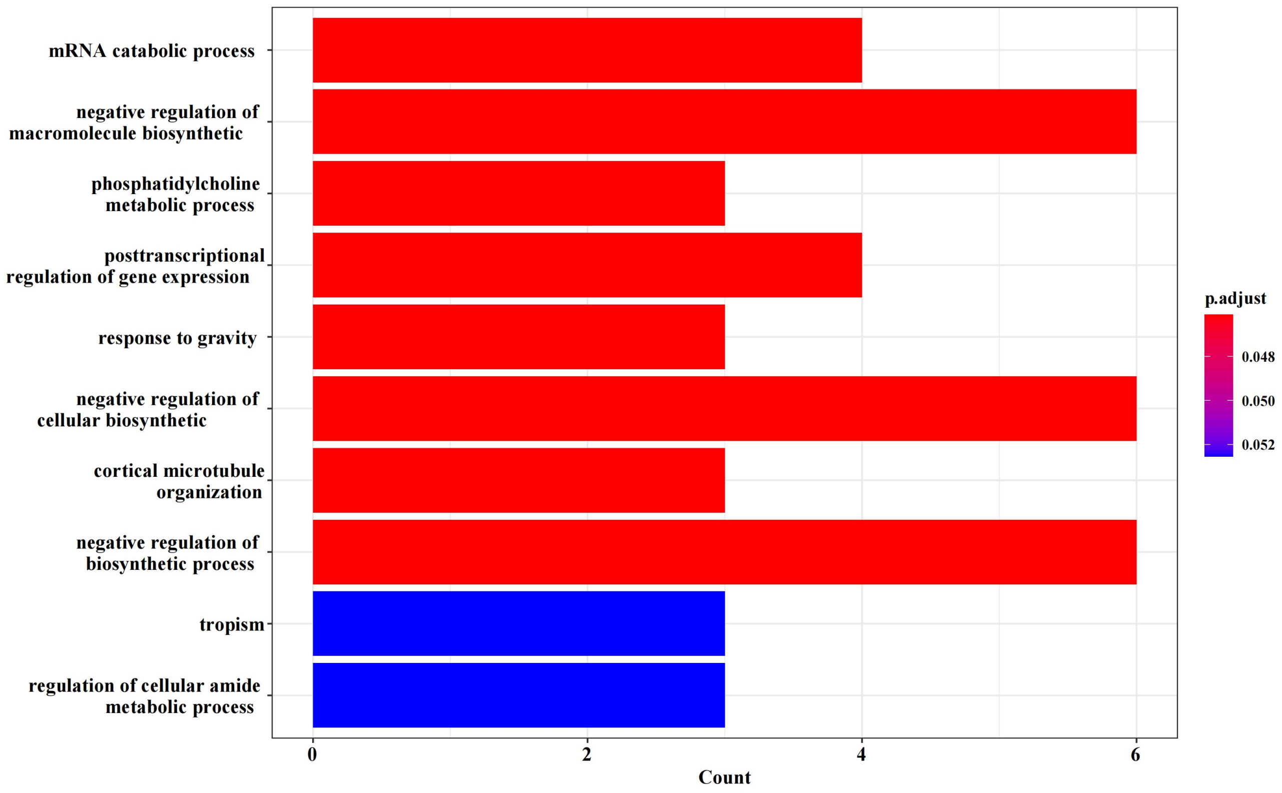

Figure S16. KEGG enrichment of DEGs in switch compartments

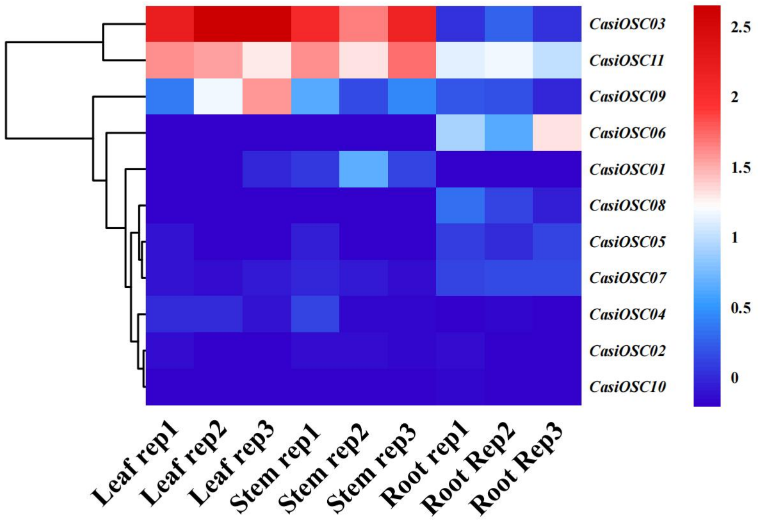

Figure S17. RNA-seq analysis of *OSC* genes in different tissues.

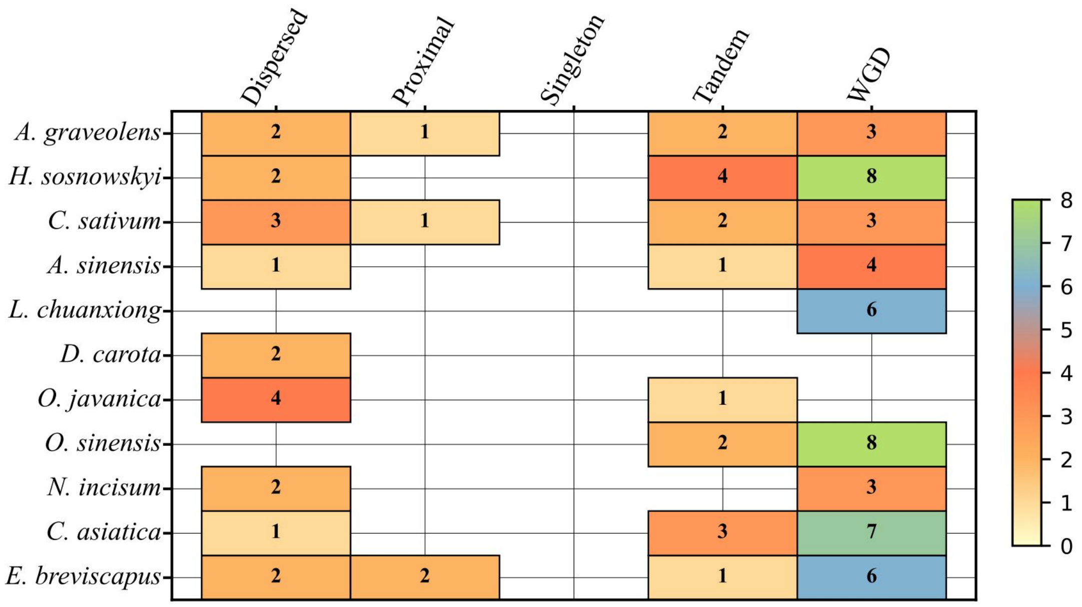

Figure S18. Duplicates classification of *OSC* gene family in 11 plants.

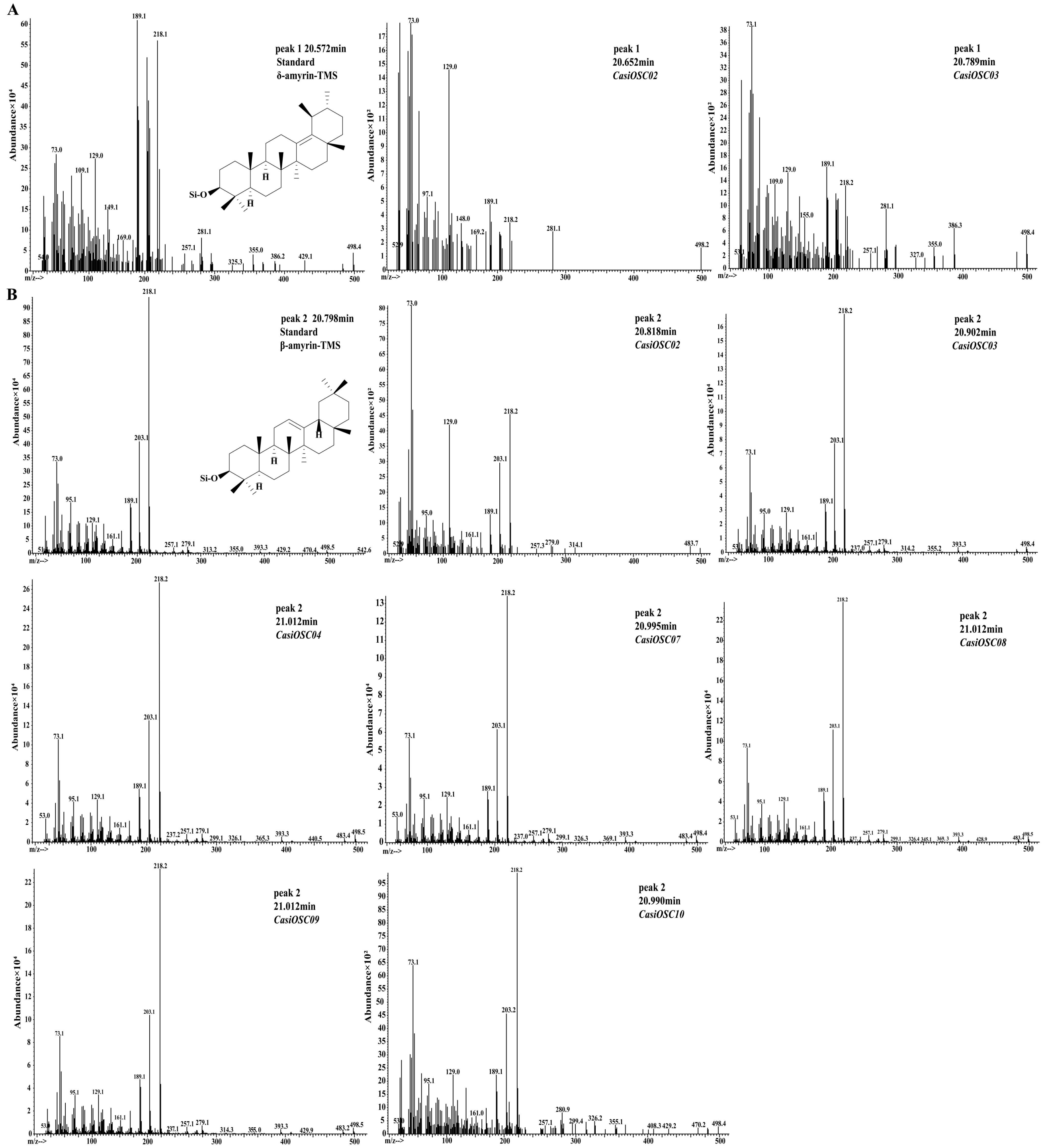

Figure S19 Mass spectra identification for compound 1 (δ-amyrin) and compound 2 (β-amyrin).

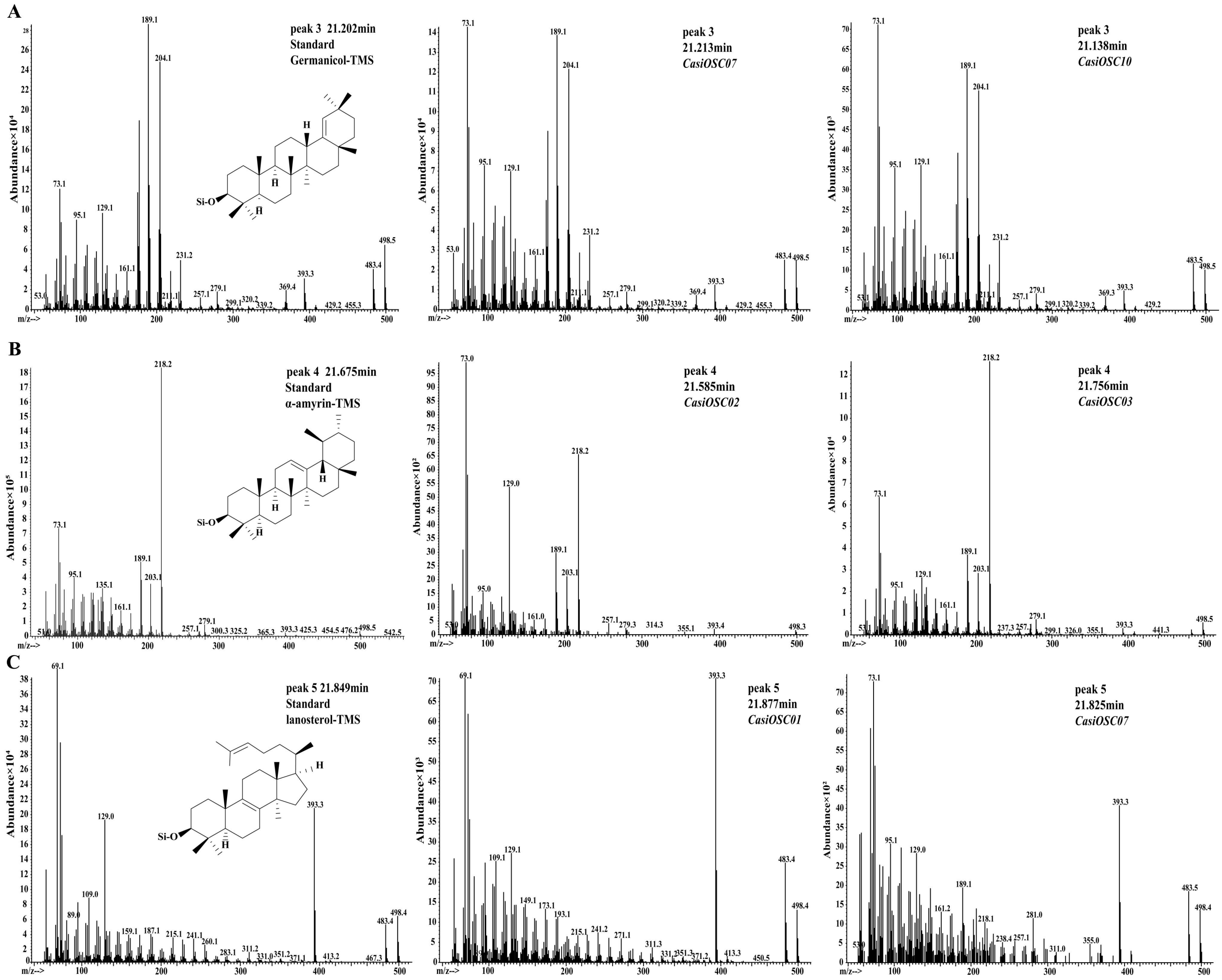

Figure S20 Mass spectra identification for compound 3 (Germanicol) and compound 4 (α-amyrin), compound 5 (lanosterol).

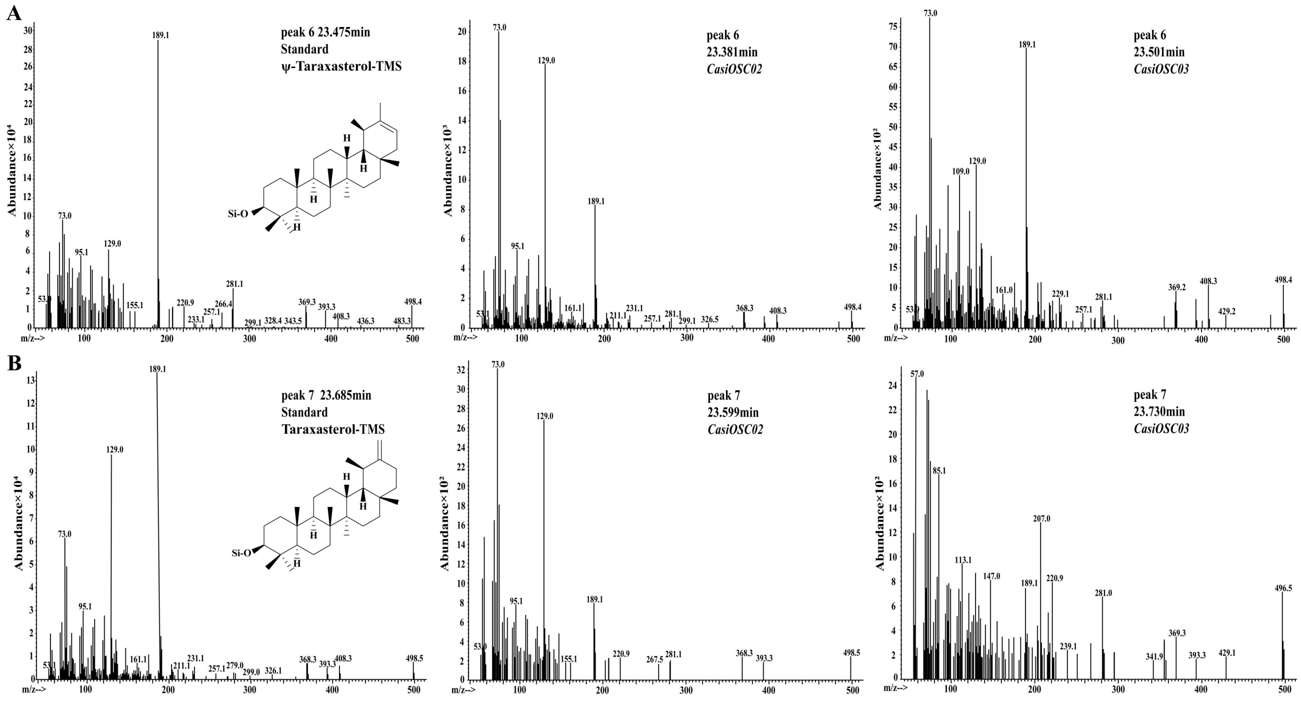

Figure S21 Mass spectra identification for compound 6 (ψ-taraxasterol) and compound 7 (taraxasterol).

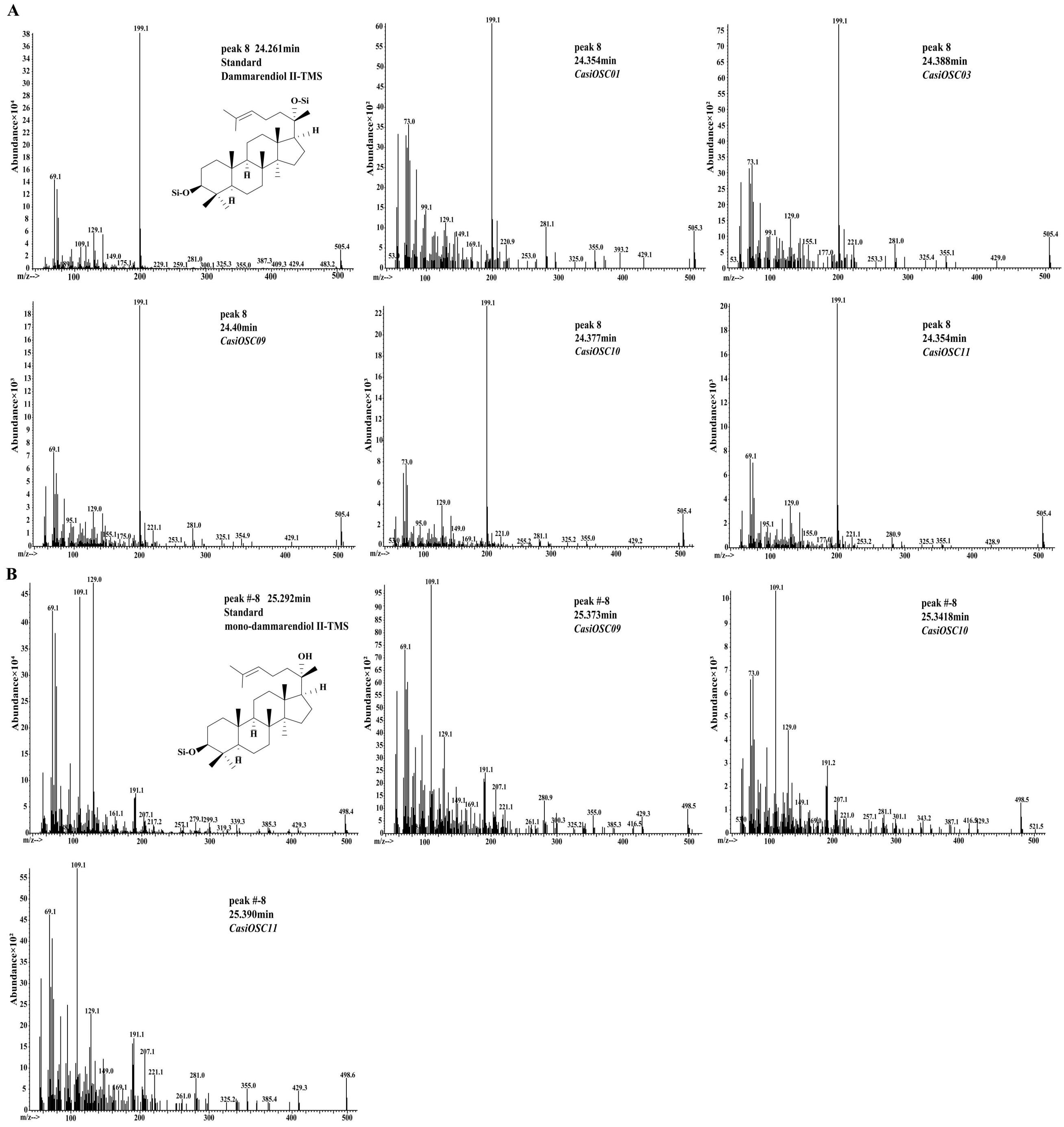

Figure S22 Mass spectra identification for compound 8 (dammarendiol-II). The hash (#) in the total ion chromatograms (TICs) represent dammarenediol-II mono-trimethylsily.

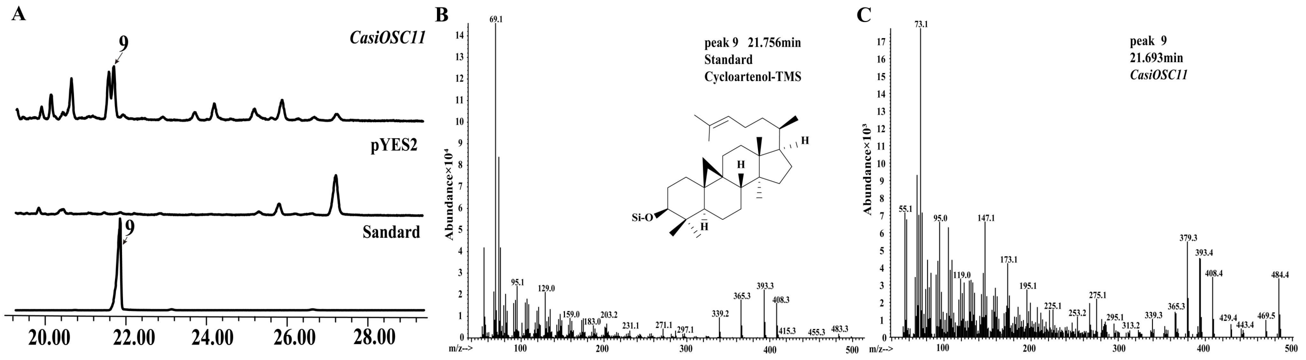

Figure S23 Functional characterization of *CasiOSC11* using heterologous expression. Mass spectra identification for compound 9 (cycloartenol). B and C are standard and *CasiOSC11*, respectively.

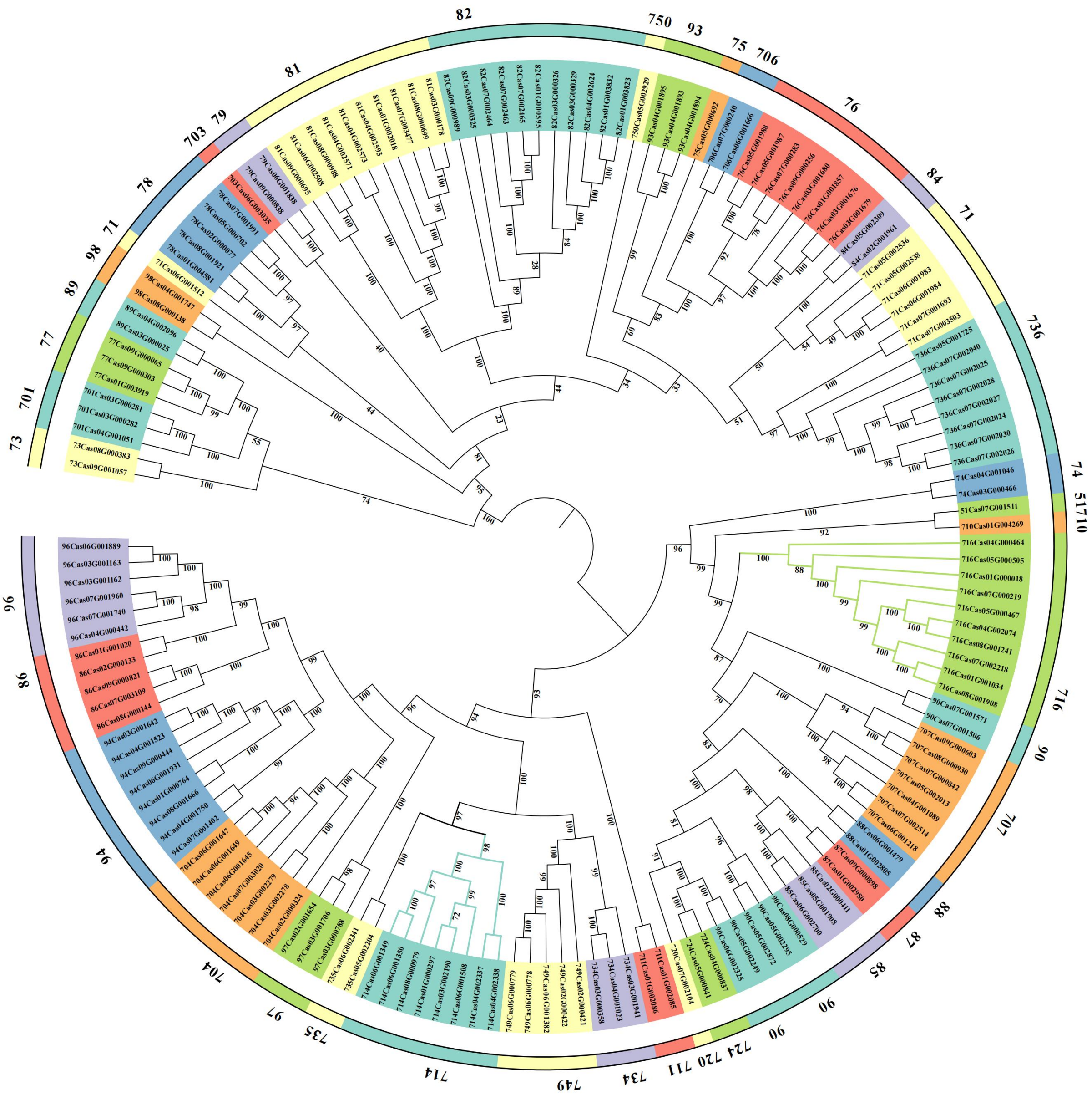

Figure 24. Phylogenetic analysis of *C. asiatica* CYP family. The numbers on the branches represent bootstrap values and the labeling in the outer ring represent subfamily name.

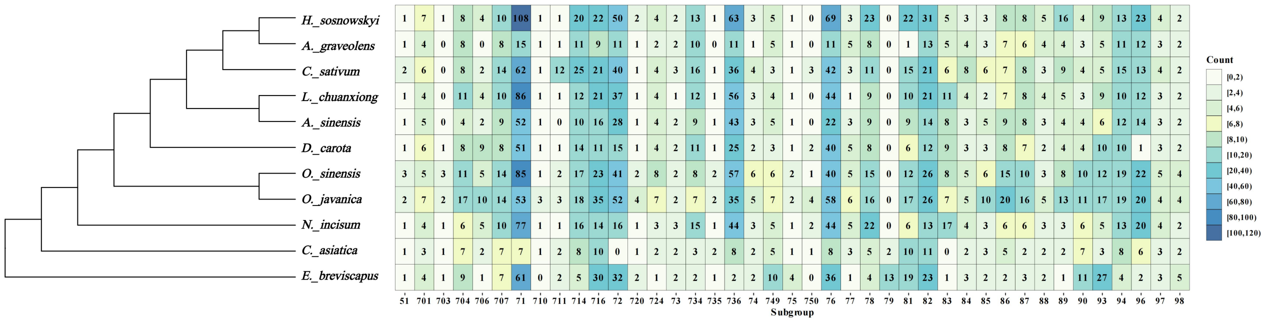

Figure S25. Phylogenomic analysis of the all the *CYP* genes in the 11 plant species.The horizontal axis represents the different subfamilies and the number of each cell in the heat map represents the number of genes contained in the corresponding subfamily of the species, which matches the legend, and visualizes the different numbers according to the color from light to dark.

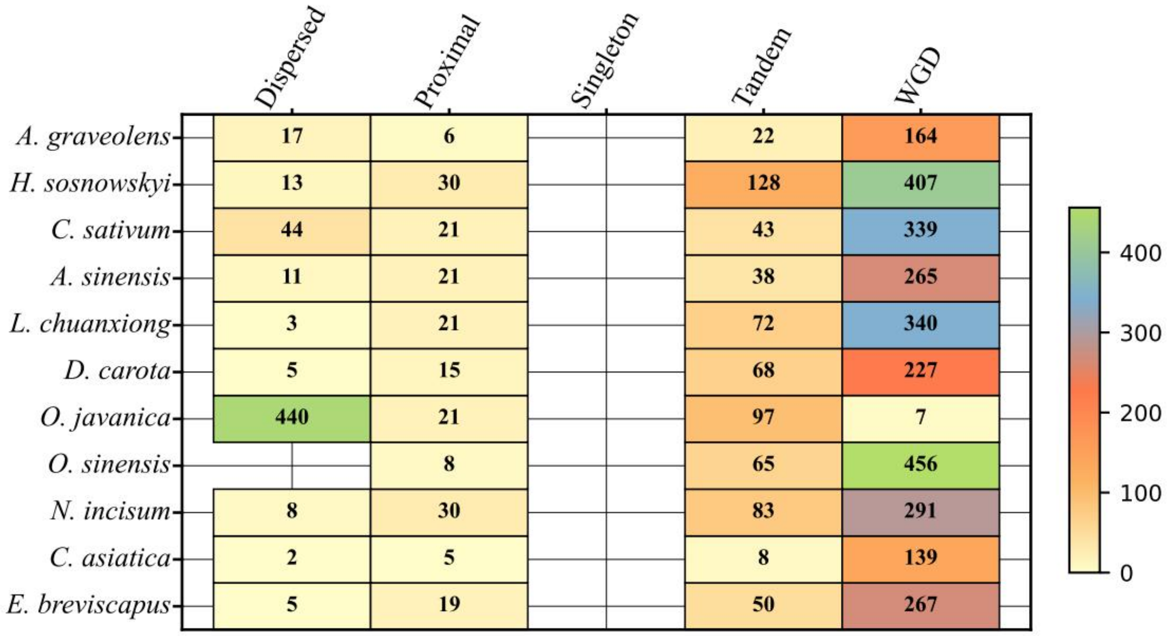

Figure S26. Duplicate classification of *CYP450* gene family in 11 plants. The *Oenanthe javanica* is not a chromosome-level genome and has a limited number of collinear gene pairs.

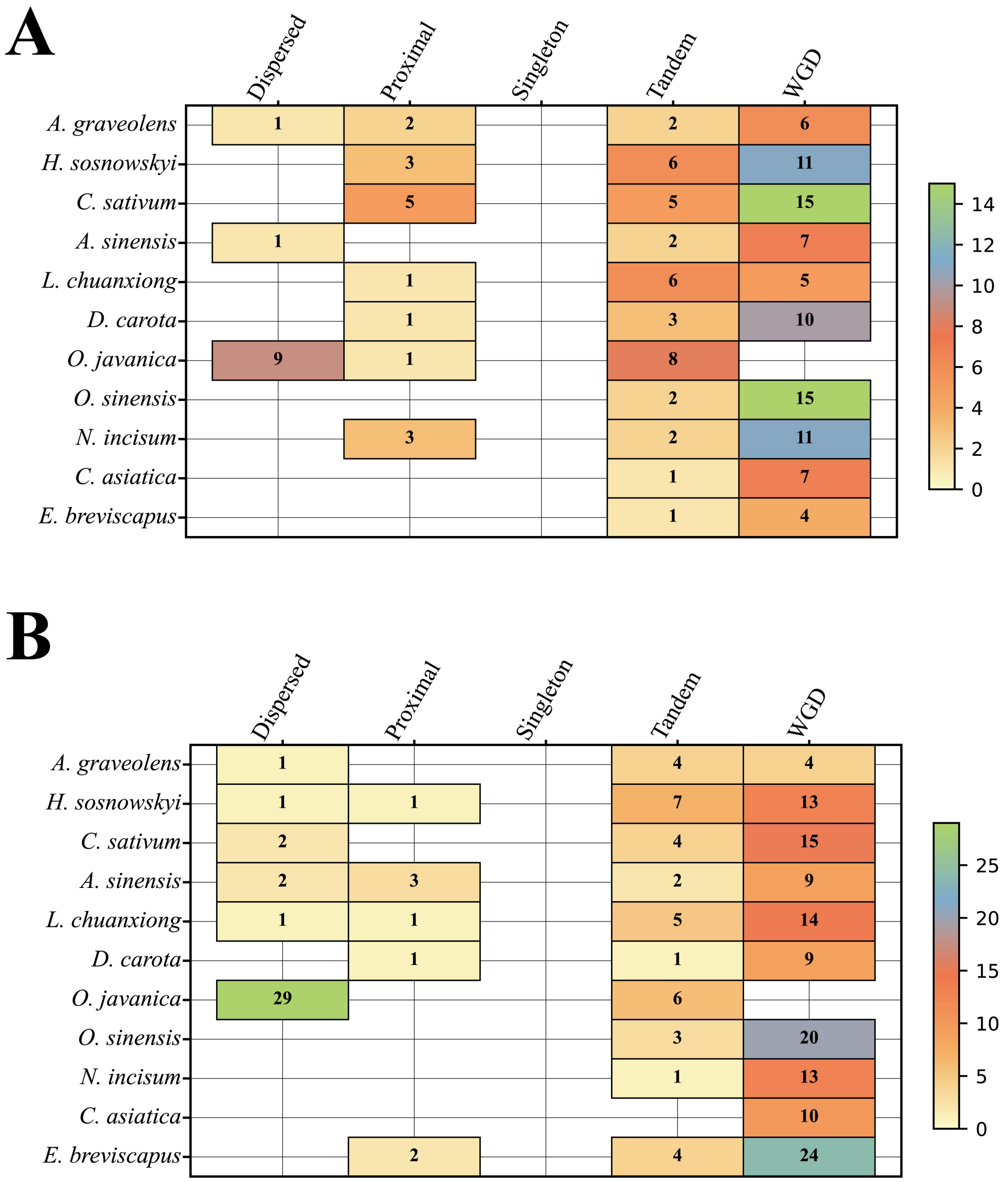

Figure S27. *CYP714* and *CYP716* gene duplication classification in 11 plants.

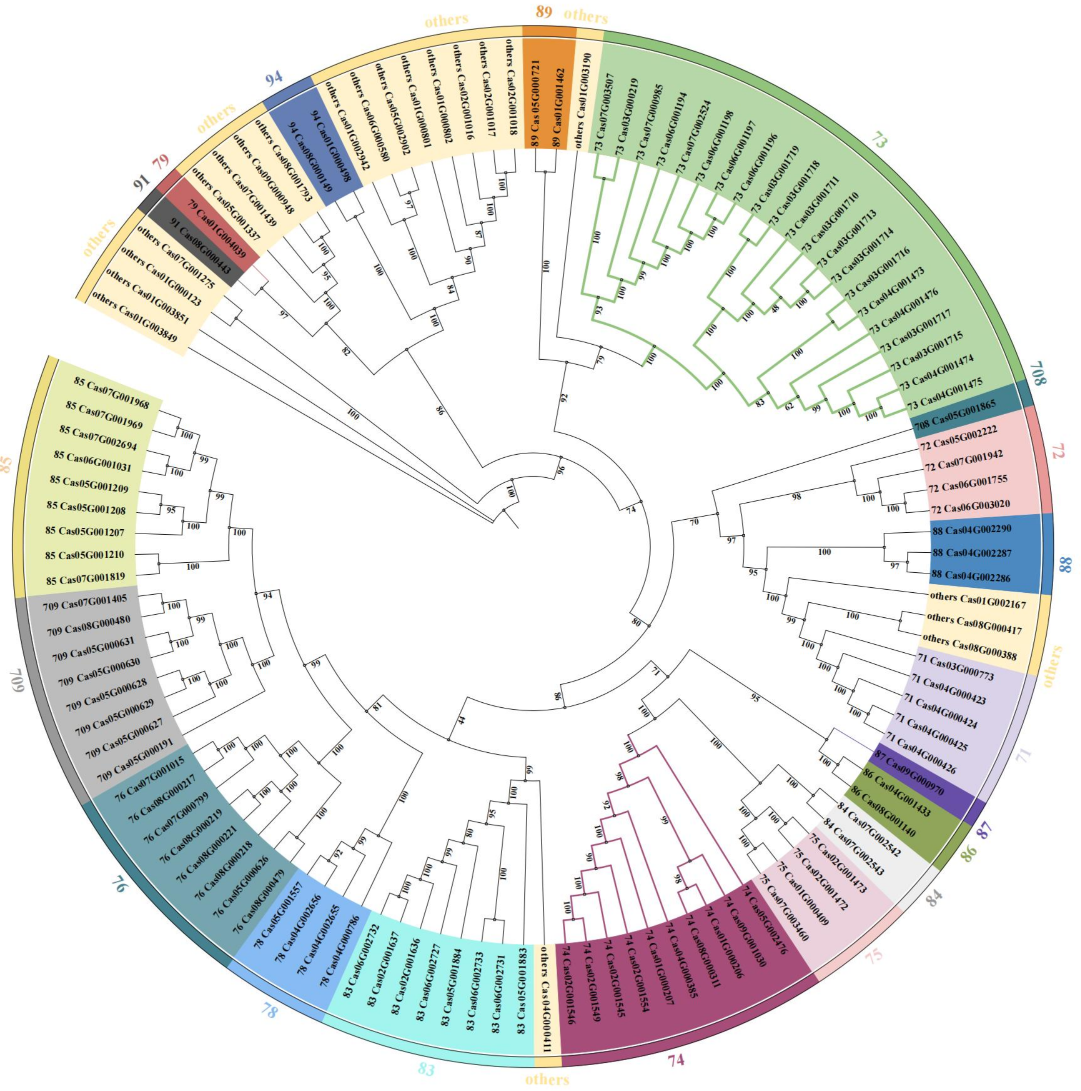

Figure S28. Phylogenetic analysis of *C. asiatica* UGT family. The numbers on the branches represent bootstrap values and the labeling in the outer ring represent subfamily name.

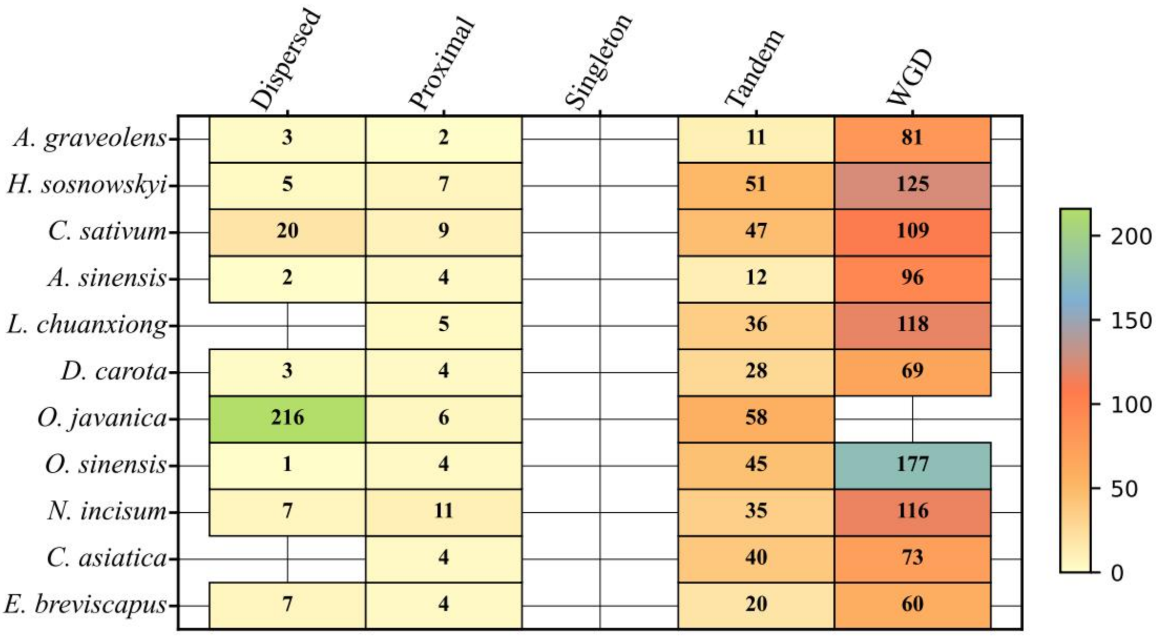

Figure S29. *UGT* gene duplication classification in 11 plants. The horizontal axis represents 5 different replication types, the vertical axis represents species, and different color gradients are used to represent the number of genes contained in the corresponding duplicated types of each species.

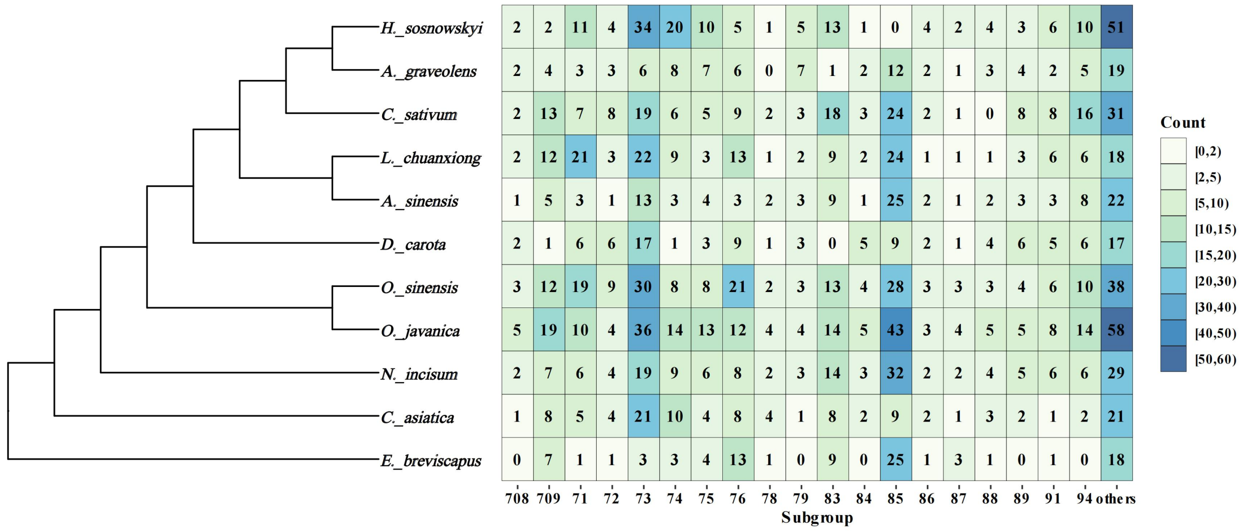

Figure S30. Phylogenomic analysis of the all the *UGT* genes in the 11 plant species. The horizontal axis represents the different subfamilies and the number of each cell in the heat map represents the number of genes contained in the corresponding subfamily of the species, which matches the legend, and visualizes the different numbers according to the color from light to dark.

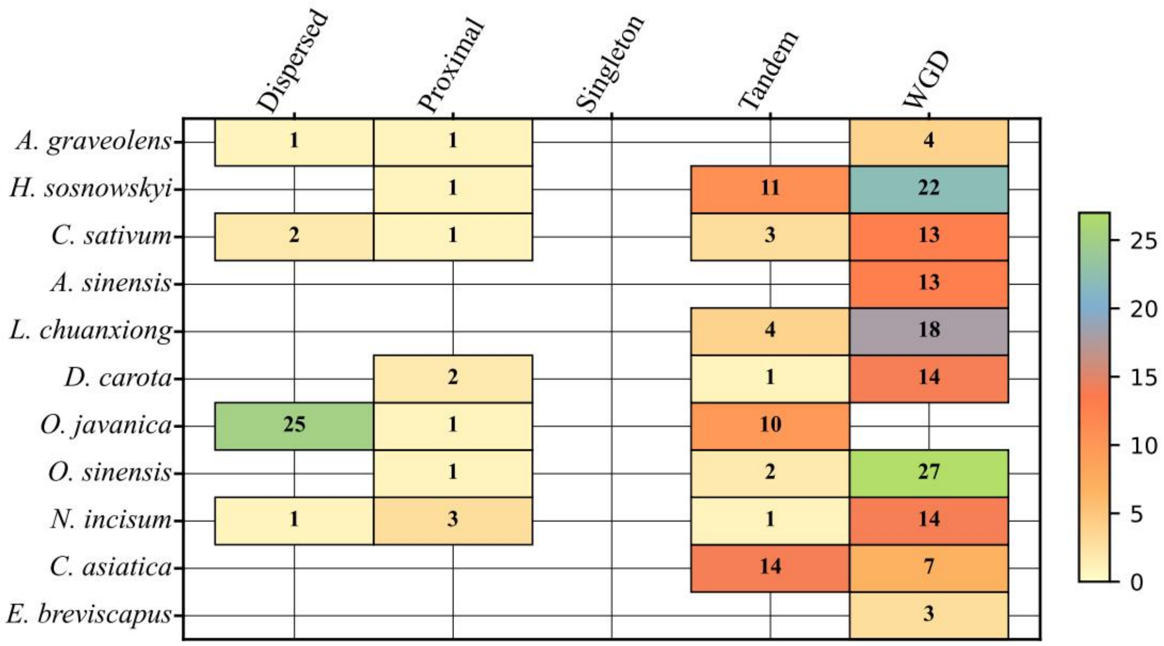

Figure S31. Gene duplication classification for *UGT73* gene subfamily in 11 plants.

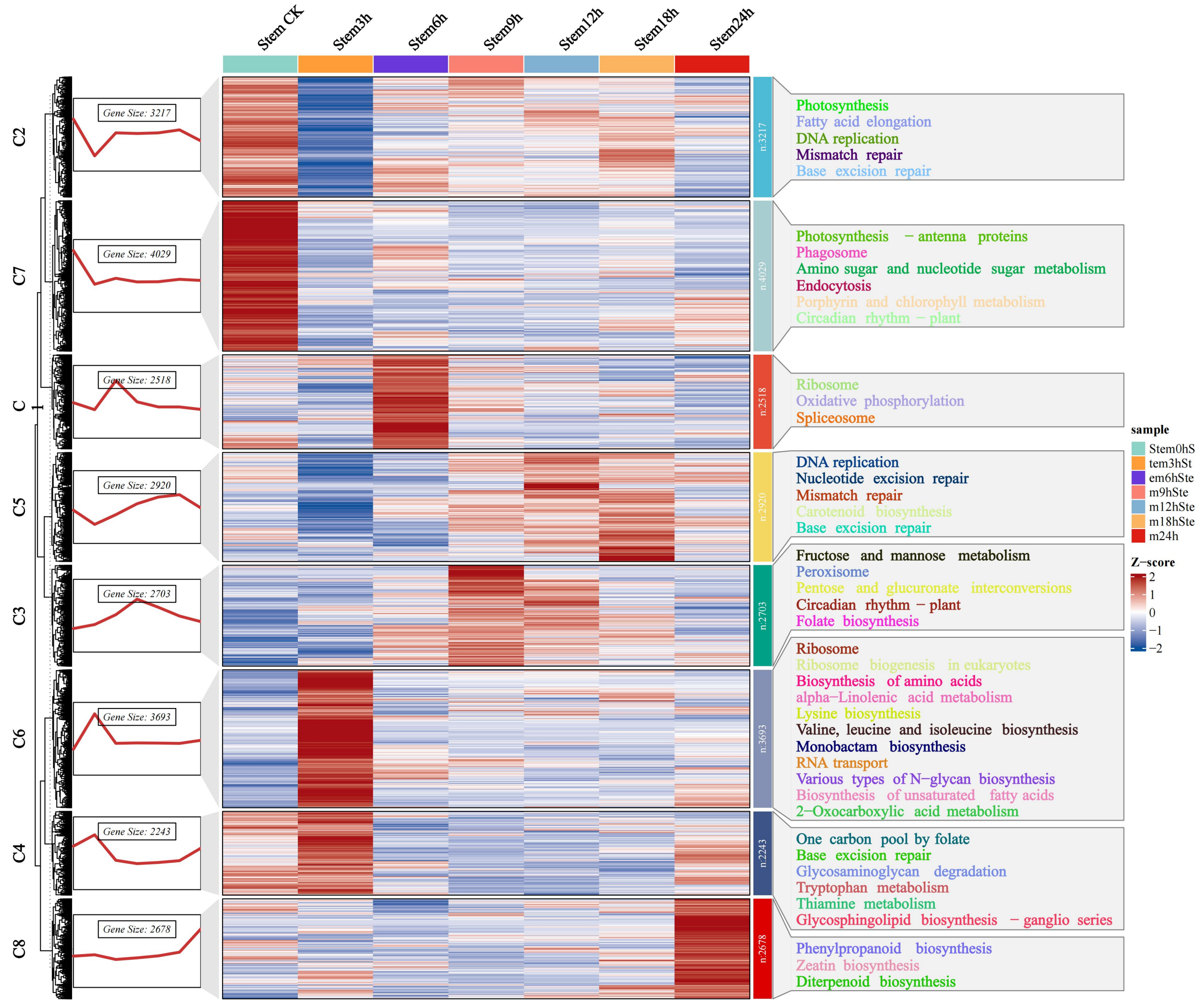

Figure S32. Gene clusters C1-C8 identified through Mfuzz analysis stem transcriptomes at time points 0,3, 6, 9, 12, 18, and 24 hours under MeJA treatment. The enrichment KEGG pathways for each cluster were annotated based on a significance level of $p < 0.05$

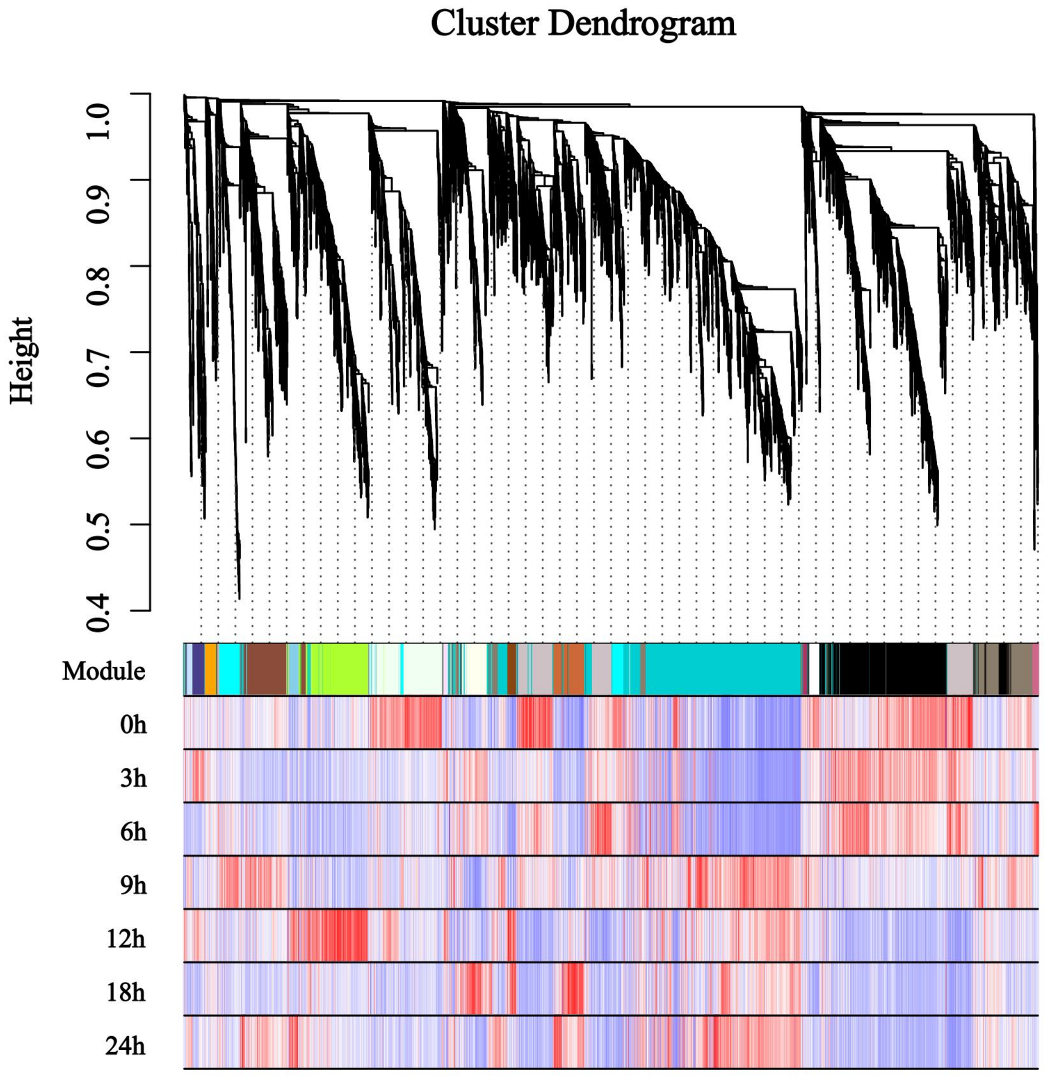

Figure S33. Gene dendrogram indicating identified gene modules and the genes associated with these modules.
